# In Vivo Imaging of Hierarchical Spatiotemporal Activation of Caspase-8 during Apoptosis

**DOI:** 10.1371/journal.pone.0050218

**Published:** 2012-11-21

**Authors:** Katsuya Kominami, Takeharu Nagai, Tatsuya Sawasaki, Yuki Tsujimura, Kenta Yashima, Yasuhiro Sunaga, Masateru Tsuchimochi, Jun Nishimura, Kumiko Chiba, Jun Nakabayashi, Koji Koyamada, Yaeta Endo, Hideo Yokota, Atsushi Miyawaki, Noboru Manabe, Kazuhiro Sakamaki

**Affiliations:** 1 Department of Animal Development and Physiology, Kyoto University, Kyoto, Japan; 2 Laboratory for Cell Function Dynamics, Brain Science Institute, RIKEN, Wako, Saitama, Japan; 3 Laboratory for Nanosystems Physiology, Research Institute for Electronic Science, Hokkaido University, Sapporo, Hokkaido, Japan; 4 Cell-Free Science and Technology Research Center, Ehime University, Matsuyama, Ehime, Japan; 5 Bio-research Infrastructure Construction Team, Advanced Science Institute, RIKEN, Wako, Saitama, Japan; 6 Meiji Institute for Advanced Study of Mathematical Sciences, Meiji University, Kawasaki, Kanagawa, Japan; 7 Cell Scale Team, Computational Science Research Program, RIKEN, Wako, Saitama, Japan; 8 Department of Electrical Engineering, Kyoto University, Kyoto, Japan; 9 Department of Immunology, Yokohama City University, Yokohama, Kanagawa, Japan; 10 Institute for the Promotion of Excellence in High Education, Kyoto University, Kyoto, Japan; 11 Research Unit for Animal Life Sciences, Animal Resource Science Center, The University of Tokyo, Kasama, Ibaraki, Japan; University of Massachusetts Medical School, United States of America

## Abstract

**Background:**

Activation of caspases is crucial for the execution of apoptosis. Although the caspase cascade associated with activation of the initiator caspase-8 (CASP8) has been investigated in molecular and biochemical detail, the dynamics of CASP8 activation are not fully understood.

**Methodology/Principal Findings:**

We have established a biosensor based on fluorescence resonance energy transfer (FRET) for visualizing apoptotic signals associated with CASP8 activation at the single-cell level. Our dual FRET (dual-FRET) system, comprising a triple fusion fluorescent protein, enabled us to simultaneously monitor the activation of CASP8 and its downstream effector, caspase-3 (CASP3) in single live cells. With the dual-FRET-based biosensor, we detected distinct activation patterns of CASP8 and CASP3 in response to various apoptotic stimuli in mammalian cells, resulting in the positive feedback amplification of CASP8 activation. We reproduced these observations by *in vitro* reconstitution of the cascade, with a recombinant protein mixture that included procaspases. Furthermore, using a plasma membrane-bound FRET-based biosensor, we captured the spatiotemporal dynamics of CASP8 activation by the diffusion process, suggesting the focal activation of CASP8 is sufficient to propagate apoptotic signals through death receptors.

**Conclusions:**

Our new FRET-based system visualized the activation process of both initiator and effector caspases in a single apoptotic cell and also elucidated the necessity of an amplification loop for full activation of CASP8.

## Introduction

Apoptosis is essential for embryonic development and the maintenance of tissue homeostasis in adults. The execution of apoptosis is mediated through the members of the caspase family [1]–[3]. In healthy cells, caspases are synthesized as zymogens containing a prodomain. Upon apoptotic stimulation, the prodomain is removed and the catalytic domain is further processed into large and small subunits resulting in production of the active form. Based on their protein structures and functions, caspases were divided into two groups: the initiator (or apical) caspases such as caspases-2, -8, -9, and -10 and the effector (or downstream) caspases including caspases-3, -6, and -7. The initiator caspases proteolytically autoactivate themselves in association with apoptotic stimuli; they then process and activate the downstream effector caspases in a proteolytic cascade. The effector caspases in turn cleave many diverse substrates, ultimately causing cell death. In mammals, it is well known that apoptosis is mediated by two major signaling pathways [4]. One of these, the extrinsic apoptotic pathway, is initiated by binding of cell surface “death receptors” such as Fas. In this pathway, apoptotic signals are mediated through the caspase cascade in association with the activation of the initiator caspase-8 (CASP8) and effector caspases such as caspase-3 (CASP3) and caspase-6 (CASP6). In the intrinsic pathway, cytochrome c is released from mitochondria by apoptotic stimuli, resulting in the activation of initiator caspase-9 (CASP9).

FRET-based detection of molecular dynamics is an elegant way to analyze protein-protein interactions during signal transduction in living cells (see review [5]). FRET studies using molecules tagged with mutant derivatives of the green fluorescent protein (GFP) have demonstrated the formation of complexes of signaling proteins in various intracellular components. So far, many groups have reported construction of biosensors for the detection of caspase activity [5]. For example, FRET-based reporters, which consist of two fluorescent proteins such as cyan fluorescent protein (CFP) and yellow fluorescent protein (YFP) and carry the caspase recognition sequence in the linker portion, have been designed and used to detect CASP3 activation. In these systems, cleavage of the linker portion by activated caspases eliminated FRET because of the physical dissociation of two fluorophores [5]. A similar FRET-based probe for CASP8 has also been generated [6]–[9]. However, it has not yet been possible to simultaneously measure the activities of both initiator and effector caspases with FRET-based reporters.

Although the caspase cascade associated with the activation of CASP8 has been investigated in considerable molecular and biochemical detail, the dynamics of CASP8 activation are not fully defined. In addition, the role of positive feedback in CASP8 activation has not yet been carefully evaluated *in vivo*, because its biological significance remains unclear. In order to address these issues, we developed a new system that simultaneously detects activation of multiple caspases, using a FRET-based biosensor. This system has the advantage of being able to monitor the dynamics of both CASP8 and CASP3 in single cells, resulting in differential activation profiles. By monitoring CASP8 activation in cells subjected to a local apoptotic stimulus, we also investigated how apoptotic signals are spatiotemporally transmitted within the cell. Using a novel cell-free system, furthermore, we performed *in vitro* reconstitution of the caspase cascade in order to confirm the *in vivo* results.

## Results

### Generation of a FRET-based Biosensor, CYR83 Consisting of Three-fluorophores with Caspase Recognition Sequences

**Figure 1:**
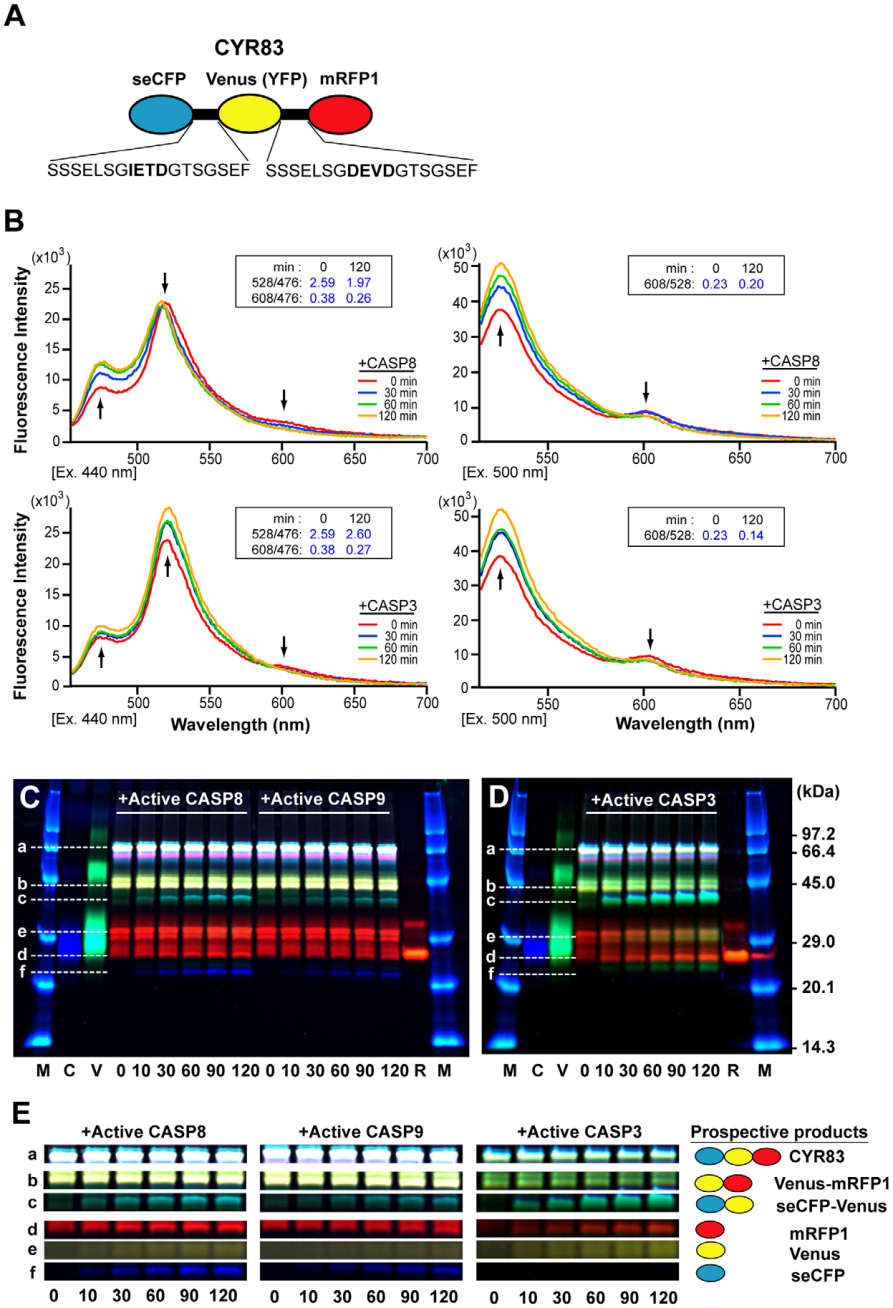
Characterization of the dual-FRET biosensor, CYR83. (**A**) A schematic structure of a FRET-based biosensor, CYR83. CYR83 consists of three fluorescent proteins, seCFP (CFP), Venus and mRFP1 (RFP); the linker portion contains two distinct caspase cleavage sequences, IETD and DEVD. (**B**) Induction of a conformational change of CYR83 by active caspases. The emission spectra of CYR83 were measured by exciting at 440 nm (left panels) or 500 nm (right panels) with a spectrophotometer before or at indicated time of incubation with recombinant active CASP8 (upper panels) or active CASP3 (lower panels). Arrows indicate the major peaks of fluorescent proteins (seCFP, Venus and mRFP1), and the direction reveals the dynamics of the fluorescence intensity. The numbers in the inset box indicate the emission ratio on 528 nm/476 nm and 608 nm/476 nm or 608 nm/528 nm before and after 120 min of incubation with active caspases. (**C**, **D**) *In vitro* cleavage assay of CYR83 by active caspases. Fluorescence data shown in panels of Figure S1A–F were merged and represented as (C) and (D). Lower-case characters indicate full length CYR83 (a) and the cleaved peptide fragments (b-f) during incubation with active caspases. Numbers indicate incubation time. Abbreviations; M, FITC-conjugated molecular weight markers; C, seCFP; V, Venus; R, mRFP1. (**E**) Profiles of the products cleaved by active caspases. Fluorescent bands corresponding to the processed seCFP, Venus, mRFP1, seCFP-Venus and Venus-mRFP1 and the intact CYR83 were extracted from the fluorescence images shown in (C) and (D).

To simultaneously monitor the dynamics of both initiator and effector caspase activation in the single cells undergoing apoptosis, we developed a dual-FRET system. This system is based on the use of a fusion protein, CYR83, which consists of three fluorescent proteins, super-enhanced CFP (seCFP), Venus (a variant of YFP) [10] and monomeric red fluorescent protein 1 (mRFP1) [11]. The linker portion contains two distinct caspase cleavage sequences, IETD and DEVD (Figure 1A). Therefore, CYR83 was expected to be suitable as a FRET-based biosensor for the detection of both CASP8 and CASP3 activation.

To define the FRET efficiency from seCFP to Venus or from Venus to mRFP1, we examined recombinant CYR83 proteins purified from cell lysates of transfected E. coli. First we analyzed the fluorescence spectrum of recombinant CYR83 by exciting in a fluorometer at either 440 nm or 500 nm (Figure 1B). FRET efficiently occurred from seCFP to Venus or from Venus to mRFP1, as can be seen from the appearance of main emission peaks of 528 nm (Figure 1B, left panels) or 608 nm (Figure 1B, right panels), specific for Venus or mRFP1, respectively. Importantly, a small mRFP1-specific peak was also detected by exciting at 440 nm (Figure 1B, left panels). This characteristic indicated that the energy transfer took place from seCFP to mRFP1, either passing through Venus or directly. Next, we examined whether the FRET efficiency in the mixture changes during incubation with either active CASP8 or CASP3 proteins. In the presence of recombinant active CASP8, the fluorescence intensity at 476 nm (seCFP peak) increased while the Venus peak decreased when the mixture was excited at 440 nm, resulting in a decrease of the emission ratio of 528 nm/476 nm from 2.59 to 1.97 after 2 h incubation (Figure 1B, upper left panel). This dynamics of the FRET efficiency was caused by physical dissociation between seCFP and Venus due to cleavage. In addition, the fluorescence intensity at the mRFP1 peak also decreased during reaction. This was confirmed by detecting an increase of the peak at 528 nm with a concomitant decrease of the fluorescence intensity at 608 nm when excited at 500 nm (Figure 1B, upper right panel), suggesting the additive cleavage of the linker portion between Venus and mRFP1 by CASP8. These data suggest that the lowering of the FRET efficiency from seCFP to Venus/mRFP1 occurred as a consequence of the processing of CYR83 by active CASP8. In the presence of recombinant active CASP3, the DEVD site between Venus and mRFP1 was preferentially processed during incubation. This was judged from the result: an increase of the peak at 528 nm and a decrease of the peak at 608 nm by exciting at 500 nm, leading to a decrease of the emission ratio (from 0.23 to 0.14) after 2 h incubation (Figure 1B, lower right panel). Unexpectedly, a Venus-specific peak also increased when the mixture was excited at 440 nm, resulting in a slight increase of the emission ratio, 2.59→2.60 (Figure 1B, lower left panel). This phenomenon was assumed to be “de-quenching” resulting from the elimination of energy transfer from seCFP to mRFP1. Taken together, these data suggest that CYR83 is a FRET-based biosensor molecule capable of distinguishing the distinct proteolytic activities of multiple caspases.

### Target Specificity of Caspases to CYR83

The substrate specificities of caspase family proteases have been reported [12]–[14]. CASP3 and caspase-7 (CASP7) prefer DEVD; CASP6 prefers VEAD; CASP8 prefers (I/L)ETD; and CASP9 prefers LEHD in the intramolecular portion of substrates. We also examined the activities of these caspases on recombinant CYR83, by *in vitro* cleavage assay. The products of CYR83 processing by active caspases were separated by SDS-PAGE and detected by scanning of fluorescence in a gel. Several cleaved peptide fragments of CYR83 proteins emerged in a time-dependent manner in mixtures that contained active caspases (Figure S1A–F, shown by arrows). By merging fluorescence images specific for seCFP, Venus and mRFP1 (Figure 1C, 1D), we showed that each active caspase yields a distinct processing pattern for CYR83. Corresponding with the data shown in Figure 1B, active CASP8 mainly cleaved at the IETD sequence in the linker portion, resulting in the time-dependent appearance of two cleaved peptide fragments, seCFP and Venus-mRFP1 (Figure 1C, 1E). As expected, CASP9 recognized and cleaved the same target sequences as CASP8 did (Figure 1C, 1E), though (measuring by units) it was only half as efficient (Figure S2A). Furthermore, by estimating the concentration of CASP8 and CASP9, we found 25-fold more CASP9 than CASP8 is required to achieve the same rate of CYR83 cleavage, indicating that CASP8 has a much higher processing activity than CASP9 with respect to CYR83. Both initiator caspases were also capable of processing another caspase recognition sequence, DEVD, in CYR83, resulting in a small amount of both seCFP-Venus and mRFP1 products (Figure 1C, 1E). These data indicated that CASP8 and CASP9 can specifically recognize the target sequence of CYR83, but more active molecules of CASP9 are required to cleave the target substrate *in vitro*. Moreover, we estimated both Vmax and Km values for active CASP8, calculating Vmax = 0.90 (ng/U/min) and Km = 690 (nM) (Figure S2B, S2C). These values are comparable to those of other caspases examined [12]. Among the effector caspases, active CASP3 preferentially recognized and cleaved the DEVD sequence, but rarely showed activity against the IETD site, as shown in Figure 1B. The processed mRFP1 and seCFP-Venus fragments were mainly detected in time-dependent manner (Figure 1D, 1E). Thus, we confirmed distinct substrate preference of active caspases for recombinant CYR83; the data also suggest that the decrease of the emission ratio of the FRET efficiency of CYR83 occurs in proportion to caspase activity *in vitro*.

### Real-time Imaging of the Dynamics and Patterning of Both CASP8 and CASP3 in the Single Cells

To examine whether microscopic analysis can be used to detect dual-FRET at the single-cell level, we expressed CYR83 in HeLa cells and examined the fluorescence intensity during apoptosis. Our five-filter channel method (see Materials and Methods) allowed acquisition of images of each of the three fluorophores and two “raw” FRET images in living cells. By setting a small spot on the edge of cells and monitoring its fluorescence, we could detect the rapid drop of the fluorescence intensity due to cell shrinkage (Figure S3). This event is helpful to determine time zero as when dying cells were shifted off the spot. It also enabled us to standardize the time schedules of individual cell progressing through apoptosis, allowing us to graphically compare of caspase activities.

**Figure 2:**
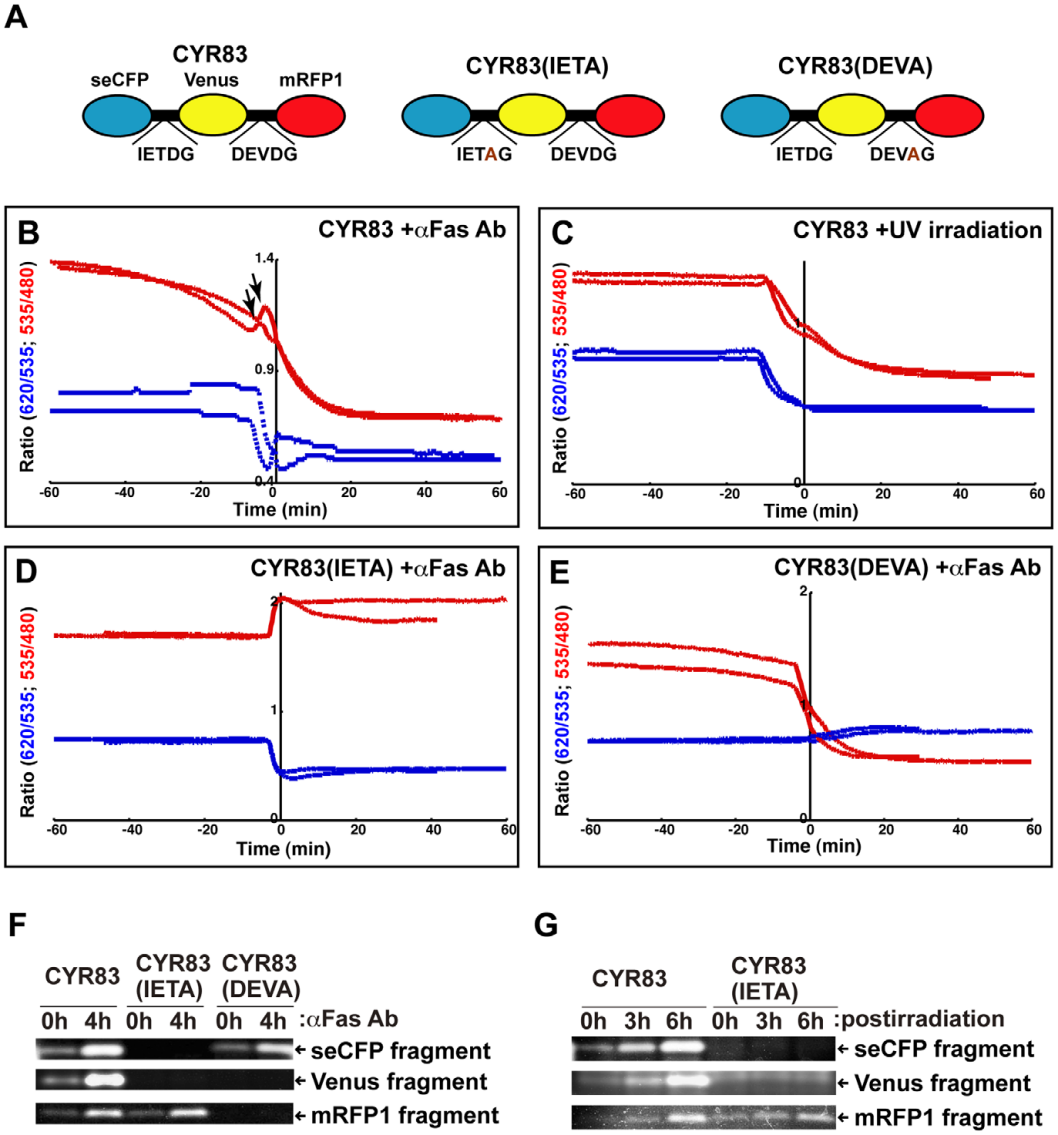
Monitoring of caspase activation with the CYR83 in single cells. (**A**) A schematic structure of CYR83 and its variants. In CYR83(IETA) variant, the IETD sequence was replaced by IETA; in the CYR83(DEVA) variant, DEVD was replaced by DEVA. (**B**) The graphic pattern of the emission ratio based on the fluorescence intensity of the CYR83 in single cells undergoing apoptosis. The CYR83-expressing HeLa cells were induced to undergo apoptosis with an agonistic anti-Fas antibody and monitored by dual-FRET. As shown in Figure S3, time course was set up before and after 1 h of cell shrinkage. The temporal fluctuations of the emission ratio on Venus/seCFP and mRFP1/Venus in single cells are plotted as red and blue lines, respectively. The IETDase and DEVDase activities are inversely proportional to graphic data. The arrow indicates a rebound detected by monitoring the fluorescence. (**C**) The CYR83-expressing HeLa cells were induced to undergo apoptosis by UV-irradiation and monitored by dual-FRET. A time course of the emission ratio is indicated. (**D**, **E**) HeLa cells expressing CYR83 variants were monitored for fluorescence. Transfected cells expressing CYR83(IETA) (D) or CYR83(DEVA) (E) were treated with an anti-Fas antibody and monitored for fluorescence. **(F**, **G)** Fluorescence image analyses on the proteolytic processing profiles of CYR83 and its variants. HeLa cells expressing CYR83 or its variants were subjected to extrinsic (F) and intrinsic (G) apoptotic stimuli at indicated times. Cell extracts prepared from those cells were resolved by SDS-PAGE and scanned for fluorescence in the gel using the imaging analyzer. Among fluorescence bands detected in panels of Figure S5A–C and S5E–G, three bands corresponding to each seCFP, Venus and mRFP1 peptide fragments were chosen and represented as (F) and (G).

We monitored fluorescence in CYR83-expressing single cells after Fas ligation, converted to a movie indicating the ratio images of FRET (Movie S1), and presented its serial images as a time course (Figure S4). These data clearly showed that the emission ratio of FRET on Venus/seCFP and mRFP1/Venus is differentially changed during apoptosis. To define the dynamics of proteolytic activity of CASP8 and CASP3 for CYR83 in single cells, we examined the graphic pattern indicating the ratio changes of dual-FRET (Figure 2). The decrease of the emission ratio inversely corresponded to the increase of caspase activity. We found stepwise activation of CASP8 in single cells; the first phase occurred approximately 30 min prior to cell shrinkage and the second phase started at minus 5 min and continued until it reached the plateau (Figure 2B, shown by red lines). To determine whether both phases of CASP8 activation uniquely occurred through the extrinsic signaling pathway, we examined cell death induced by ultraviolet (UV)-irradiation. In cells undergoing apoptosis through the intrinsic pathway, only the second phase of CASP8 activation was observed (Figure 2C, shown by red lines). This data argues that the first phase of CASP8 activation detected by treatment with an anti-Fas antibody is unique to death receptor-mediated apoptosis. As proposed in the previous report [15], a small amount of active CASP8 seems to be sufficient to transmit Fas-mediated apoptotic signals. Our system made it practicable to monitor these signals at the single cell level. By comparison of the patterns of CASP8 and CASP3 activation, we found that CASP3 activation occurs prior to the second phase of CASP8 activation in both the extrinsic and intrinsic apoptotic pathways; furthermore, the activation pattern is exponential (Figure 2B, C, shown by blue lines). Interestingly, we reproducibly detected a rebound that occurred at the time between the first and second phases of CASP8 activation (Figure 2B, shown by arrows). We speculated that this event is due to earlier cleavage of CYR83 by activated CASP3, and reflects the loss of energy transfer from seCFP to mRFP1 and concomitantly increasing the fluorescence intensity of Venus, which proves “de-quenching”. To confirm this interpretation, we generated two types of CYR83 variants, CYR83(IETA) and CYR83(DEVA) (Figure 2A). CYR83(IETA) is a variant in which the IETD sequence is replaced by IETA, resulting in no cleavage between seCFP and Venus by CASP8. By referring to the data shown in Figure 1B, it was expected that the emission ratio of Venus/seCFP would inversely increase when the linker portion between Venus and mRFP1 of CYR83(IETA) is cleaved by CASP3. Indeed, the experimental data validated our hypothesis, showing a steady state increase of the FRET ratio for Venus/seCFP, but not mRFP1/Venus, in CYR83(IETA)-expressing cells (Figure 2D). This phenomenon elucidated the appearance of the rebound detected by monitoring the original CYR83. In the case of CYR83(DEVA), in which DEVD was replaced by DEVA, the pattern of the FRET ratio on CASP8 activation was similar to that of CYR83, except that no rebound was observed (Figure 2E).

To validate the results obtained with the cytological image analyses, the proteolytic processing patterns of CYR83 and its variants CYR83(IETA) and CYR83(DEVA) in HeLa cells were biochemically examined after extrinsic or intrinsic apoptotic stimuli (Figure 2F, 2G). Fluorescent bands corresponding to the cleaved seCFP, Venus, mRFP1, seCFP-Venus and Venus-mRFP1 peptide fragments were detected in cell extracts from CYR83-expressing cells after a 4-h incubation with an anti-Fas antibody (Figure 2F, Figure S5A–C). However, in CYR83(IETA)-expressing cells, fluorescent bands corresponding to the seCFP and Venus peptide fragments, but not mRFP1, were not detected, suggesting no further processing of seCFP-Venus (Figure 2F and Figure S5A–C). In the case of CYR83(DEVA)-expressing cells, the cleavage in the linker between Venus and mRFP1 did not occur, resulting in no Venus and mRFP1 peptide fragments (Figure 2F and Figure S5A–C). In contrast, an endogenous CASP3-specific substrate, poly(ADP-ribose) polymerase (PARP) [16] was equivalently cleaved in three different types of transfected cells (Figure S5D). After UV-irradiation, we also examined the processing patterns of CYR83 and CYR83(IETA) (Figure 2G, Figure S5E–G). They were completely coincident with those of cells subjected to extrinsic stimuli. That is, the processed seCFP, Venus and mRFP1 peptide fragments were detected in CYR83-expressing cells in a time-dependent manner. In the case of CYR83(IETA)-expressing cells, only a mRFP1 peptide fragment, but not Venus and mRFP1 peptide fragments was apparent and increased during incubation. However, there was no difference in the processing rate of endogenous PARP between these transfected cells (Figure S5H). Consequently, these results indicated that the dynamics of the FRET ratio change is directly associated with cleavage of biosensors. Thus, we established a system able to simultaneously monitor the dynamics of both initiator and effector caspase activation in single cells, and to present real-time images of the intracellular events that occur during apoptosis. Using this system, we were able to verify distinct activation patterns for CASP8 and CASP3.

### Reconstitution of the Caspase Cascade in vitro, Leading to the Sequential Activation of Caspases

**Figure 3:**
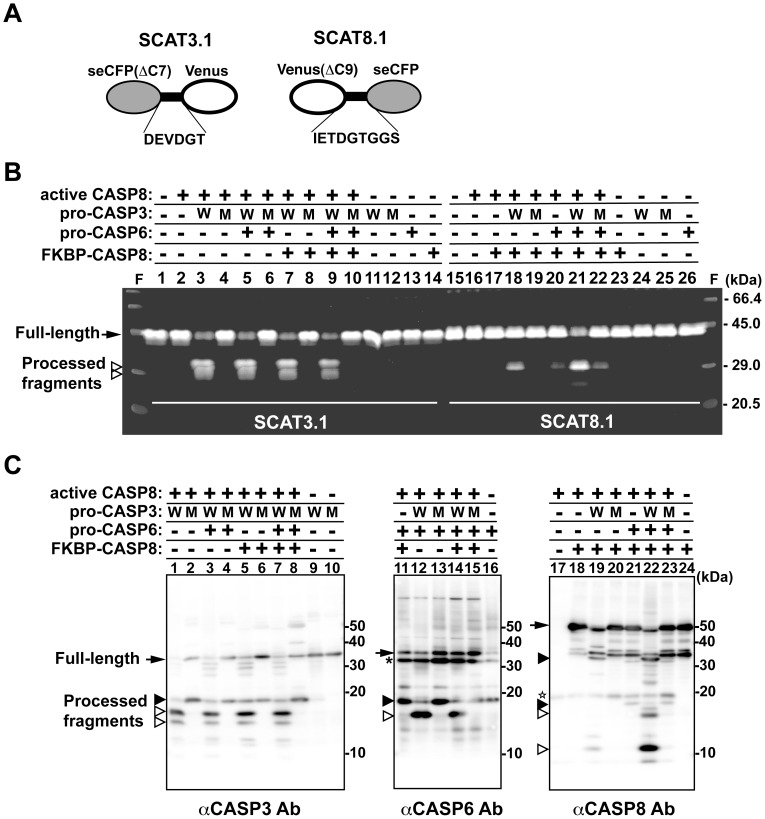
Functional reconstitution of the caspase cascade *in vitro*. (**A**) A schematic structure of FRET-based probes SCAT3.1 and SCAT8.1. Both SCAT3.1 and SCAT8.1 are fusion molecules consisting of seCFP and Venus and containing either the CASP3 recognition sequence (DEVD) or the CASP8 recognition sequence (IETD) in the linker portion. (**B**) *In vitro* cleavage assay of the FRET-based probes with recombinant proteins. Various reaction mixtures containing either SCAT3.1 or SCAT8.1 combined with several caspases were incubated at 37°C for 2 h, and the cleaved products were analyzed by SDS-PAGE followed by scanning of the fluorescence in the gel. The arrow and arrowheads indicate the full-length and cleaved form of the probes, respectively. (**C**) Immunoblot analyses for monitoring the processing profile of procaspases in the reaction mixtures. After incubation for 2 h, the processing pattern of procaspases in the mixture was analyzed by SDS-PAGE following immunoblotting with antibodies against CASP3, CASP6 or CASP8. The arrow indicates the full-length procaspase and the white and black arrowheads indicate the completely and partially cleaved fragments, respectively. The star indicates active CASP8 included in the mixture in advance and the asterisk shows a non-specific reaction. Abbreviations: W, wild-type pro-CASP3; M, the protease-defect pro-CASP3 mutant.

By single-cell assays with the dual-FRET system, we detected two phases of CASP8 activation via the extrinsic pathway. The second phase of CASP8 activation was thought to be dependent on the positive feedback amplification loop in the apoptotic pathway. To test this hypothesis, we investigated the caspase cascade *in vitro* using recombinant caspases and fluorescent substrates. We failed to produce procaspase-3 (pro-CASP3) and procaspase-6 (pro-CASP6) in *E. coli* because these caspases were naturally converted into active forms without apoptotic stimulation (data not shown). Therefore, we produced recombinant proteins pro-CASP3, pro-CASP3 mutant, pro-CASP6 and FKBP-CASP8 in a cell-free protein synthesis system [17]. FKBP-CASP8 protein is a chimeric protein composed of the binding domain of FK506-binding protein (FKBP) and the protease domain of CASP8 [18]. The previous report showed that conversion to the active form by autoprocessing occurs only when this molecule is dimerized by a synthetic divalent FKBP ligand. This property excludes the possibility of autoactivation by self-oligomerization. As a substrate, we also prepared two recombinant molecules, SCAT3.1 and SCAT8.1. SCAT3.1 carries the DEVD sequence in the linker portion between seCFP and Venus while SCAT8.1 has the IETD sequence between Venus and seCFP (Figure 3A). These molecules are also useful as a FRET-based probe, as with CYR83 [19], [20].

We performed *in vitro* reconstitution of the caspase cascade by mixing active CASP8 and procaspases together and assessing the activity of the cascade by examining processed SCAT3.1 and SCAT8.1 (Figure 3B, lanes 1–14 and lanes 15–26). As shown in Figure 1, recombinant active CASP8 can cleave the caspase target sequences of CYR83 *in vitro*. In this case, 1 unit was required for the complete cleavage of the IETD sequence in CYR83 within 2 h. For *in vitro* reconstitution, we used 6.25**×**10^−2^ units of active CASP8, resulting in no cleavage of substrates within 3 h (Figure 3B, lanes 2 and 16). Mixture of pro-CASP3 with active CASP8 led to the cleavage of SCAT3.1, indicating the conversion of pro-CASP3 to an active form and the acquisition of the ability to cleave substrates (Figure 3B, lanes 3 and 11). This was confirmed by detection of a processed active form (large subunit) of CASP3 by immunoblot analysis (Figure 3C, lanes 1 and 9). Active CASP8 alone or in combination with mutant CASP3, whose catalytic activity is defective, was insufficient to cleave SCAT3.1 (Figure 3B, lanes 4 and 12). Addition of pro-CASP6 and/or FKBP-CASP8 did not affect the processing of SCAT3.1 (Figure 3B, lanes 5–10, 13, 14). On the other hand, the IETDase activity against SCAT8.1 was scarcely detected in the presence of active CASP8 and pro-CASP3 (Figure S6, lanes 2, 4, 5). After further addition of FKBP-CASP8 into the mixture, a cleaved fragment of SCAT8.1 was detected (Figure 3B, lane 18). Addition of pro-CASP6 into a mixture that included active CASP8, pro-CASP3 and FKBP-CASP8 enhanced the IETDase activity against SCAT8.1, resulting in a 2.5-fold increase in the processed form (Figure 3B, lane 21). In the mixture, pro-CASP6 was cleaved only when pro-CASP3 is converted to an active form (Figure 3C, lanes 3, 4, 12 and 13), suggesting acquisition of the protease activity of CASP6 in cooperation with active CASP3. Although FKBP-CASP8 alone or with active CASP8 has no protease activity (Figure 3B, lanes 17 and 23), this molecule was converted to an active form due to processing by other caspases included in the mixture (Figure 3C, lanes 18–24) and then possessed the ability to cleave SCAT8.1. Additionally, there was only slight processing of SCAT8.1 in the mixture except FKBP-CASP8 (Figure S6, lanes 10–13). These data indicate that the amplitude of CASP8 activation in the mixture occurs by a hierarchical proteolytic process. Furthermore, we have reconstituted the *in vitro* caspase cascade, including the conversion of pro-CASP8 to an active form through positive feedback amplification.

### Examination of the Effects of the CASP8 Downregulation on a Feedback Loop

**Figure 4:**
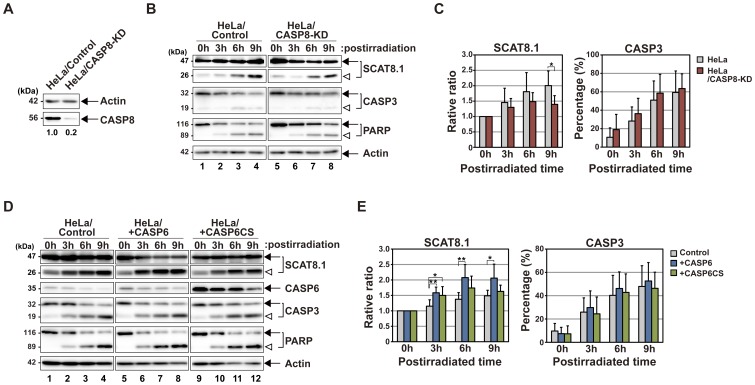
Effects of the downregulation of CASP8 on the processing of the SCAT8.1 biosensor. (**A**) Immunoblot analyses of CASP8 proteins downregulated by RNA interference. Cell extracts from HeLa cells carrying either pCSIIU6Tet-Neo or pCSIIU6Tet-sh*CASP8*-Neo were prepared and analyzed by SDS-PAGE, followed by immunoblotting with anti-CASP8 and anti-actin antibodies, respectively. The numbers indicate the ratio of endogenous CASP8 between two stable lines by calculating the measurements of CASP8 and actin using an image analyzer. (**B**) Immunoblot analyses of CASP8-knockdown cells subjected to intrinsic apoptotic stimuli. Cell extracts from parental HeLa cells (lanes 1–4) or HeLa/CASP8-KD cells (lanes 5–8) expressing SCAT8.1 were prepared at the indicated times after UV-irradiation. Endogenous CASP3, PARP, actin and exogenous SCAT8.1 were examined with indicated specific antibodies. A representative of four independent experiments is shown. Arrows indicate intact proteins while arrowheads identify the processed peptide fragments. (**C**) The static analysis of the immunoblot data. The relative ratio of processed peptide fragments to total SCAT8.1 proteins was estimated by measuring the intensity of immunoblot bands in four independent experiments using an image analyzer. The ratio of the processed form relative to total CASP3 proteins was also shown as percentage. The both graphs indicate the means and standard deviations of the estimated ratios. Significant differences between the two groups were evaluated by Student’s *t*-test. An asterisk shows p < 0.05. (**D**) Immunoblot analyses of HeLa cells overexpressing CASP6 or its mutant. HeLa cells were transiently transfected either with plasmids carrying CASP6 (lanes 5–8), CASP6CS (lanes 9–12) or control vector (lanes 1–4), and cell extracts were prepared at indicated times after UV-irradiation. Endogenous CASP3, PARP, actin and exogenous CASP6 and SCAT8.1 were examined with indicated specific antibodies. A representative of four independent experiments is shown. The arrow and arrowhead indicate the full-length and the cleaved form of proteins examined, respectively. (**E**) The static analysis of the immunoblot data. Both ratio and percentage of processed fragments to total SCAT8.1 and processed CASP3 to total CASP3 were estimated and shown as the bar graph. Statistical validation was performed by Student’s *t*-test. *p < 0.05, **p < 0.01.

We indicated that the full activation of CASP8 occurs through a positive feedback loop both in cells and *in vitro*. To confirm the reliability of our verification, we tested whether the downregulation of CASP8 affects the proteolytic processing of molecules, which are specifically recognized by active CASP8. For this examination, we established the HeLa stable cell line, HeLa/CASP8-KD, in which CASP8 transcripts are downregulated by RNA interference (Figure 4A). CASP8 expression in HeLa/CASP8-KD cells was reduced to 20% at the protein level. Therefore, CASP8 activation via a feedback loop was expected to decline in this cell line subjected to intrinsic apoptotic stimuli, leading to the insufficient processing of CASP8-specific substrates. In SCAT8.1-expressing HeLa/CASP8-KD cells, the cleavage of the biosensor was controlled compared with that of SCAT8.1-expressing HeLa cells after UV-irradiation (Figure 4B, 4C). However, endogenous CASP3 and PARP were equivalently cleaved in these transfected cells (Figure 4B, 4C), indicating that the apoptotic signal cascade is normally intact in HeLa/CASP8-KD cells with the exception of CASP8 activation. These results support the notion that CASP8 is directly involved in the processing of SCAT8.1 in cells. As shown in Figure 3, we showed the CASP6 activation is required for the full activation of CASP8. In the related study, we also showed the overexpression of wild-type CASP6 promotes the proteolytic processing of CASP8 in HeLa cells by Fas ligation whereas the overexpression of either a protease-deficient mutant, CASP6CS or a constitutively phosphorylated mutant, CASP6SE retards the CASP8 cleavage [20]. These observations suggest that the regulation of CASP6 activity indirectly affects the processing of SCAT8.1 by CASP8. We examined the processing patterns of SCAT8.1 in HeLa cells overexpressing wild-type CASP6 or CASP6CS after UV-irradiation (Figure 4D, 4E). Excessive expression of wild-type CASP6 promoted the processing of SCAT8.1. In contrast, overexpression of a protease deficient mutant, CASP6CS did not promote the processing of SCAT8.1. Endogenous CASP3 and PARP were cleaved in these transfected cells without significant differences (Figure 4D, 4E). Consequently, our results suggest that CASP8, which is processed downstream of CASP3 and CASP6, becomes an active form, leading to the enhanced cleavage of CASP8-specific target molecules. Additionally, it is possible that some other caspase is involved in the SCAT8.1 processing after UV-irradiation, because the cleavage of this probe was not dramatically decreased in CASP8 knockdown cells.

### Propagation of CASP8 Activation by the Diffusion Process

**Figure 5:**
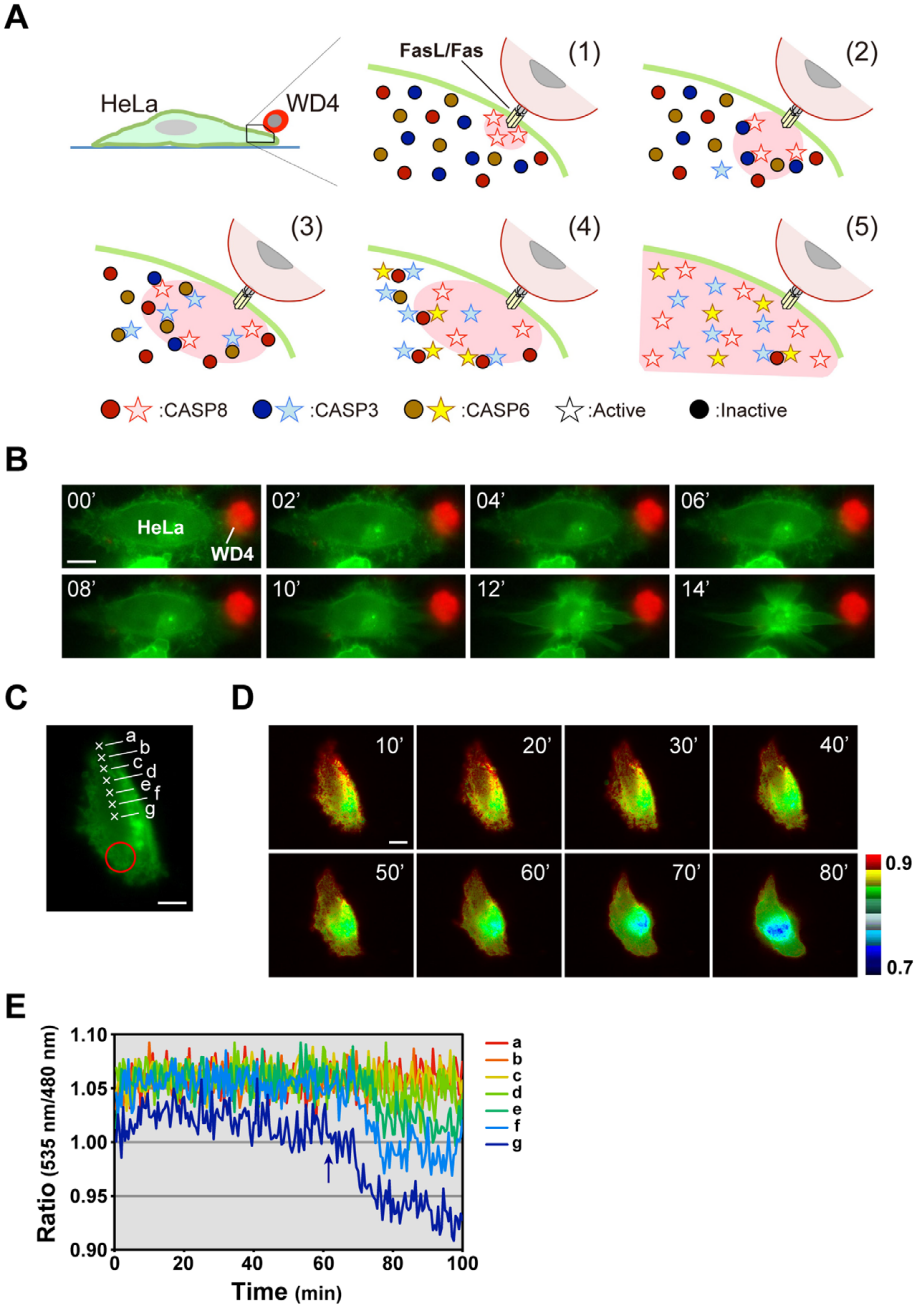
Graded activation of CASP8 in response to a focal apoptotic stimulus. (**A**) A schematic illustration indicating the propagation of apoptotic signals associated with CASP8 activation. Through focal stimulation by cell-to-cell interactions between an effector WD4 cell (expressing FasL), and a recipient HeLa cell, CASP8 activation occurs at the DISC, and then expands to the whole cell via the caspase cascade and the reaction-diffusion system, as illustrated in the steps (1) to (5). Spreading of active CASP8 was indicated by a red zone. (**B**) Serial images of a HeLa cell undergoing apoptosis induced by cell-to-cell interactions with an effector cell. The HeLa cell (green) expressing a membrane-bound FRET-based biosensor, GsSCAT8, was induced to undergo cell death through contact with a single WD4 cell (red). Numbers indicate time after taking the first image. (**C**) A FRET image of a HeLa cell expressing GsSCAT8. A red circle indicates the position of an overlaid WD4 cell. (**D**) The dynamics of CASP8 activation in dying HeLa cells, observed using GsSCAT8. The fluorescence intensity on the cell surface of a HeLa cell shown in (C) was captured over time after overlaying a WD4 cell. Serial images are displayed using pseudo colors based on calculation of the emission ratio. Numbers indicate time after taking the first image. A representative cell from 24 cells examined in seven independent experiments is shown. Scale bars, 10 µm. (**E**) The graphic patterns of the FRET ratio on seven points marked with a cross shown in (C). An arrow indicates onset of the drop of the FRET ratio in the point “g”.

Finally, we examined how apoptotic signals perceived through death receptors propagate throughout the entire cell after focal stimulation. For this purpose, we analyzed the spatiotemporal activation pattern of CASP8 using a plasma membrane-bound SCAT8, GsSCAT8, in single cells. In the experiments presented above, we used an agonistic anti-Fas antibody as a pro-apoptotic stimulant. Under these conditions, cells are bathed in medium containing antibody and exposed to stimulants over the entire cell surface. In contrast, in living organisms, under most physiological conditions, cells expressing Fas might be stimulated locally. Therefore, as shown in Figure 5A, we used a subline of human Fas ligand (FasL)-expressing WR19L cells, WD4, as a pro-apoptotic stimulant. This cell line expresses a membrane-bound FasL, which lacks the processing site allowing release of the extracellular region [21]. Contact with WD4 cells leads to strong induction of cell death in cells expressing human Fas. Attachment of a single WD4 cell caused the HeLa cell expressing GsSCAT8 to promptly undergo cell death (Figure 5B). Fluorescence images of dying cells were taken and converted to pseudo-color images after calculation of the FRET ratio. The data indicate that CASP8 activation occurred locally in the region of contact with a WD4 cell and then expanded throughout the cell. Because the FRET ratio was radially changed from proximal to distal area of a WD4 cell attachment site, implying that CASP8 proteins dispersed in the cytoplasm are gradually activated and sequentially process GsSCAT8 bound to the cytoplasmic face of the plasma membrane (Figure 5C, 5D, Movie S2). To verify this observation, we examined the dynamic range of the FRET ratio change of GsSCAT8 at the near and far sites from the WD4 cell attachment site (Figure 5E). Among seven monitoring points, we detected the sequential decline of the FRET ratio from the near point to the far point. This data supports the spatiotemporal expansion of active CASP8. Thus, the global activation pattern of CASP8, which is illustrated in Figure 5A, was detected in all HeLa cells that we observed undergoing cell-to-cell interactions with a single WD4 cell; this pattern indicates that active caspases spatiotemporally spread through the cytosol by diffusion.

**Figure 6:**
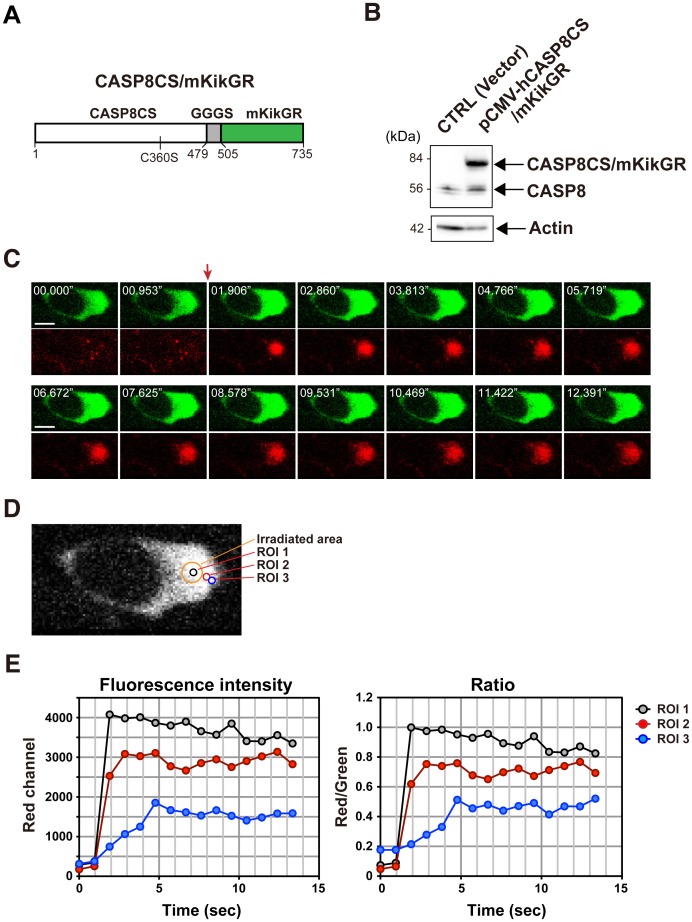
Visualization of the diffusional behavior of CASP8 fused with a photoconvertible fluorescent protein. (**A**) A schematic structure of a fusion protein, CASP8CS/mKikGR. CASP8CS/mKikGR encodes 735 amino acids and consists of human CASP8CS and a photoconvertible fluorescent protein, mKikGR, which were linked with triple GGGS repeats. CASP8CS is a mutant with a cysteine-to-serine (C360S) substitution in the active site of the protease domain. (**B**) Immunoblot analysis. Exogenous CASP8CS/mKikGR and endogenous CASP8 in cell extracts of HEK293 transfected cells were detected by SDS-PAGE, followed by immunoblotting with anti-CASP8 antibody. (**C**) Confocal images showing diffusion of photoconverted CASP8CS/mKikGR. Images for original green (upper panels) and photoconverted red (lower panels) fluorescence of CASP8CS/mKikGR were taken every 0.953 sec. A red arrow indicates the irradiation timing by 405 nm laser beam. Scale bars, 10 µm. (**D**) A magnified view of the green fluorescence image. An orange circle (diameter 4.140 µm) represents the irradiated area (IA). The black, red, and blue circles (diameter 1.656 µm) represent ROIs marked for the measurement of fluorescence intensity. ROI 1 was set in the photoconverted region and ROI 2 and 3 were placed outside of the photoconverted region. The difference between the center-to-center distance of IA-ROI 2 and IA-ROI 3 is 1.445 µm. (**E**) Time course of the photoconverted red fluorescence intensity of CASP8CS/mKikGR (left) and the red to green emission ratio (right) in the ROIs 1–3.

To further investigate the cytoplasmic behavior of CASP8, we generated a fusion protein, CASP8CS/mKikGR consisting of a protease-deficient mutant of human CASP8, CASP8CS, and a photoconvertible fluorescent protein, mKikGR (monomeric Kikume Green-Red) [22], linked with the GGGS linker, and expressed it in HeLa cells (Figure 6A, 6B). We observed that laser irradiation (405 nm) quickly change the fluorescence emission of this fusion protein from green to red in the stimulated area (Figure 6C). Sequential monitoring of both green and red fluorescence of the pre- and post-photoconversion states displayed the expansion of CASP8CS/mKikGR from the stimulated area throughout the cell. Furthermore, we measured both green and red fluorescence intensity in several regions of interest (ROIs) from near to far (Figure 6D, 6E). In ROI 2, the red fluorescence intensity was maximally increased at 2.860 sec. In ROI 3, which was set up at the 1.445 µm far side, the peak of the fluorescence intensity was detected at 4.766 sec. Based on these data, we estimated that the diffusion coefficient of the photoconverted CASP8CS/mKikGR in this transfected cell is approximately 1.096 µm^2^/s. We repeated experiments to calculate the mean of the diffusion coefficient of CASP8CS/mKikGR, resulting in 0.793±0.39 µm^2^/s (Table 1).

**Table 1:** Estimation of the diffusion coefficient of CASP8CS/mKikGR.

Cell No.	Inter-center distance(*a*) between IA and ROI 2 (µm)	Peak time *t_1_* (sec)	Inter-center distance(*b*) between IA and ROI 3 (µm)	Peak time *t_2_* (sec)	Distance difference(*b–a*) (µm)	Time lag (*t_2_*–*t_1_*) (sec)	Diffusion Coefficient (µm^2^/sec)
#1	3.014	2.860	4.459	4.766	1.445	1.906	1.096
#2	2.927	1.047	3.537	2.110	0.610	1.063	0.350
#3	2.927	1.047	4.160	3.172	1.233	2.125	0.715
#4	2.777	1.281	4.628	3.844	1.851	2.563	1.337
#5	2.777	1.281	3.817	3.844	1.040	2.563	0.422
#6	3.338	1.125	4.629	3.359	1.291	2.234	0.746
#7	3.233	1.313	4.572	3.907	1.339	2.594	0.691
#8	2.927	1.313	3.414	2.610	0.487	1.297	0.183
#9	3.152	4.813	4.628	7.219	1.476	2.406	0.905
#10	2.484	2.140	4.140	5.312	1.656	3.172	0.865
#11	2.777	2.578	4.628	6.422	1.851	3.844	0.891
#12	2.898	3.360	4.554	5.594	1.656	2.234	1.228
#13	2.484	2.593	4.140	6.500	1.656	3.907	0.702
#14	2.777	3.432	4.628	5.709	1.851	2.277	1.505
#15	2.777	3.354	3.702	6.693	0.925	3.339	0.256
Mean ± SD							**0.793 ± 0.39**

The time at which the red fluorescence intensity value rises to a maximum is defined as the peak time. IA: irradiated area.

**Figure 7:**
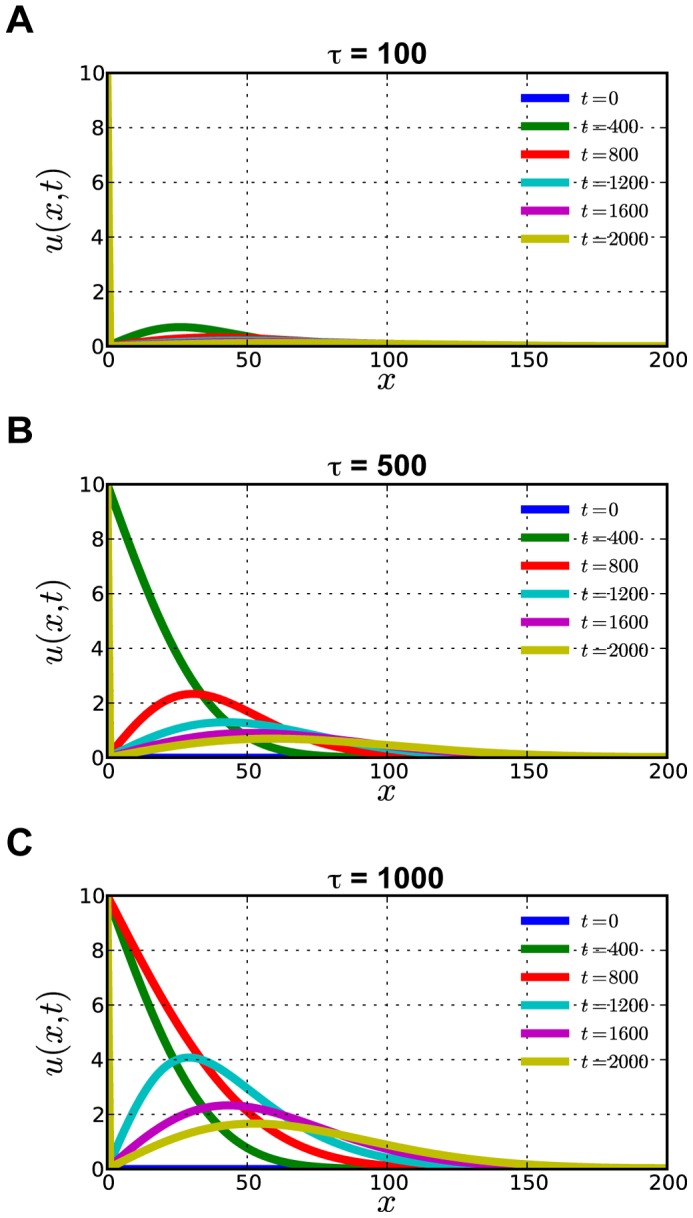
A mathematical model on the propagation of CASP8 activation. (**A**–**C**) The propagation of CASP8 activation was simulated by an one-dimensional diffusion model. The vertical axis (*u*) indicates the concentration of the activated CASP8. The horizontal axis (*x*) indicates the distance from the input source of the apoptotic signal and time zero means the starting point of the cell-cell interaction. CASP8 activation propagates from the left to the right in the graph. Each colored line in the figures gives the spatial distribution of activated CASP8 for different time, where time zero is the starting point of the cell-cell interaction. A blue line indicates the start time when the apoptotic signal is inputted. A yellow line indicates 2000 unit time that has passed from the start time. The duration (*τ*) of the input signal and was set to 100 (A), 500 (B), and 1000 (C) and *f*_0_ = 10. Here, diffusion coefficient is set to *D* = 1 and the cell size is set to *L* = 200.

The estimation of the diffusion coefficient suggests that CASP8 is able to move to the edge of the cell within several tens of seconds. However, there was a spatiotemporal lag of minutes on the mobility of active CASP8 with distance from the stimulus point (Figure 5). To explain the significant difference in timing, it is necessary to consider the reaction-diffusion system, because it was thought that CASP8 activation, which is initially occurred in the focal region, radially expands throughout the cell. Therefore, we generated a mathematical diffusion model to verify the existence of such a time lag (Figure 7). The simulation showed that CASP8 activation depends on the duration time of the extrinsic apoptotic signals. When *τ* is 100 (Figure 7A), activated CASP8 slightly accumulates at the 50 unit point (*x* = 50) distant from the signal input source until the time has passed to 800 unit (*t* = 800, red line). Active CASP8 significantly increased at the point *x* = 50 when *τ* becomes 500 (Figure 7B). In the case of *τ* is 1000 (Figure 7C), the gradient of active CASP8 becomes steep. As a result, a longer duration of input signals supplies the wave of CASP8 activation to a long way. The shorter duration was insufficient to do it even though the total amount of input signals is the same. This means that active CASP8 is slowly accumulated in the opposite side of the initial signal source by the transient input. Our one-dimensional diffusion model also suggests the apoptotic signal mediated through cell-to-cell interaction is transiently transmitted. Taken together, our results suggest that CASP8 activation fully propagates through a positive-feedback amplification loop and the diffusion process. Consequently, focal activation of CASP8 is sufficient to transmit apoptotic signals through death receptors.

## Discussion

In the present study, we have established a system for visualizing apoptotic signals at the single-cell level associated with CASP8 activation, using a FRET-based biosensor. Our results revealed differential activation patterns of CASP8 depending on various apoptotic stimuli. Specifically, CASP8 showed a two-step activation profile in response to extrinsic signals. To transmit apoptotic signals to downstream molecules, it seems to be sufficient to activate small amounts of procaspase-8 (pro-CASP8), as detected in the primary phase. The second phase of CASP8 activation occurs after CASP3 activation in both extrinsic and intrinsic pathways, indicating that the positive feedback amplification process is preserved in HeLa cells. We also showed that CASP8 is spatiotemporally activated through a diffusion process in cells subjected to the focal apoptotic stimulus. These data suggest that this amplification loop is required for complete progression of apoptosis.

The FRET system takes advantage of the detection of caspase activation at the single-cell level as reported in previous studies (see review [5]). In this study, we further developed the dual-FRET system to simultaneously detect the activation of multiple caspases using a triple fusion protein, CYR83. By monitoring the activation of both initiator and effector caspases with this system, we were able to determine the progress of the caspase cascade at the single-cell level. Based on the same idea, another group has previously generated a FRET-based indicator consisting of three fluorescent proteins to simultaneously monitor CASP3 and CASP6 activation and demonstrated the sequential activation of CASP3 and CASP6 [23]. From the graphical data with the dual-FRET system, we can clearly distinguish the two stages of CASP8 activation and the rapid activation of CASP3 between these stages. In addition, from comparison of the reaction curves, we speculate that there may be a rate-limiting reaction involved in CASP6 activation; the second phase of CASP8 activation, corresponding to the terminal reaction, does not exhibit more exponential fluctuation compared with CASP3 activation (Figure 2). This speculation was experimentally confirmed by analyzing the dynamics of CASP8 activation under the conditions that CASP6 activity was regulated [20]. Thus, the FRET system is useful not only for the real-time imaging of caspase cascade activity but also for the estimation of the kinetics of these caspases at a single cell level. This system also assists in computational modeling of the apoptotic signaling pathways [8], [20], [24].

CASP8 is proximally activated in the death-inducing signaling complex (DISC) formed by death receptor ligation [25]. Therefore, it has been proposed that dimerization of pro-CASP8 in the DISC complex is prerequisite for conversion to an active form [26], [27]. However, previous studies reported an alternative mechanism for activation of CASP8 [28], [29]. Interchain proteolysis of CASP8 is shown to be sufficient to produce an active form, implying the conversion of a monomeric pro-CASP8 to an active form due to direct processing by another caspase, but not itself. Indeed, CASP8 in etoposide-treated cells is activated in a death receptor-independent manner [29]. Pro-CASP8 is processed via CASP6, which is anteriorly processed and activated by CASP3, and becomes an active form [29]–[32]. In our in vitro reconstitution of the caspase cascade, both CASP3 and CASP6 were required for full activation of CASP8 (Figure 3), indicating that they act upstream of CASP8, as described in previous studies. On the other hand, it has been suggested by controversial evidence that the processed form of CASP8 by interchain proteolysis has no enzymatic activity [33]. However, a more recent report supports the previous argument, by showing that CASP8 is activated after proteolytic processing by cathepsin D [34]. Therefore, although there is still no evidence to clarify the molecular mechanism to induce dimer formation in the cytoplasm, which might be required for activation, it cannot exclude the possibility that the cleaved CASP8 becomes an active form at this time. To better understand the interplay between CASP8 and other caspases, future studies are required to reproduce the cellular condition in the in vitro system using caspases in a defined quantitative ratio and their native conformations.

Based on both in vitro and in vivo experiments, we clarified that full activation of CASP8 occurs by a positive feedback amplification loop. Why is full activation of CASP8 required? One possibility is that it is indispensable for the complete execution of the caspase cascade. As shown in Figure 2, however, activation of CASP3 occurs rapidly and fully in response to apoptotic signals resulting from Fas ligation, suggesting that it would be sufficient for cleavage of substrates and execution of cell death. Nonetheless, even after full activation of CASP3, most CASP8 was activated. Therefore, we sought to address the physiological role of CASP8 activation by a positive feedback pathway. Setting up a working hypothesis that CASP8 activated through this pathway recognizes and processes an undefined CASP8-specifc substrate, which leads to the promotion of apoptosis, we searched for an appropriate molecule and verified it in our related study. That is, a two-pore potassium channel has been identified as a novel substrate recognized by activated CASP8 and the processing of this protein resulted in accelerated cell shrinkage (Kominami et al., manuscript submitted). Recently, it has been shown that another channel protein, Transient Receptor Potential Melastatin-like 7 (TRPM7) is cleaved by CASP8 downstream of the mitochondria-mediated signaling pathway [35]. Since CASP8 is an initiator of the apoptotic cascade, the amount of active CASP8 needed to set the apoptotic program in motion is small [20]. However, positive feedback and propagative activation of additional CASP8 would permit the efficient cleavage of these membrane proteins. Our studies suggest that the dynamics of the CASP8 activation cascade is crucial for advancing the cell to the next stage of apoptosis.

We have previously determined the diffusion coefficient of cytoplasmic soluble molecules using fluorescent proteins [36]. The diffusion coefficient of PA-GFP (239 amino acids (aa)) and Phamret (466 aa) was measured by method based on fluorescence decay after photostimulation (FDAP) and represent approximately 23 µm2/s and 14 µm2/s, respectively. The diffusion coefficient of CASP8, which consists of 479 aa, is assumed to approximate the value of Phamret, if the precursor of CASP8, pro-CASP8, exists as a monomer in the cytosol. However, the diffusion coefficient of CASP8CS/mKikGR (735 aa) was roughly estimated as 0.793±0.39 µm2/s (Table 1). This lower value suggests that pro-CASP8 makes a complex in the cytoplasm under normal conditions, resulting in the delayed diffusion. Although it has recently been shown that CASP8 makes a complex termed “Ripoptosome” with FADD and RIP1 in the cytosol [37], [38], this complex is only formed in a stressful situation. There seems to be an unknown complex containing CASP8 distinct from the Ripoptosome. Nonetheless, it was expected that CASP8 is able to move to the edge of the cell in several tens of seconds. However, there was a spatiotemporal lag of minutes on the mobility of active CASP8 with distance from the stimulus point (Figure 5). To explain the time lag observed in our experiments, we constructed the mathematical model based on the diffusion process (Figure 7). One possibility explaining the difference in time series is the delay of the active CASP8 accumulation by transient signals from the ligand-receptor complex. We theoretically indicated that the activated CASP8 is slowly accumulated at the far site from the initial source due to the transient signal. As an apical caspase, CASP8 is known to be activated in the complex with receptors and released to the cytosol. In this situation, the DED domains of CASP8 remain and may inhibit the reuse of the death receptor for new CASP8 by masking. The recent report supported this possibility by showing the tight interaction between only the DED domain and the death receptor complex [39]. Importantly, the previous study has shown that the activation of effector caspases simultaneously occurs without a lag phase in the entire cell by using a FRET-based probe [40]. This claim is at variance with our result. In that study, the soluble TRAIL was used as a pro-apoptotic stimulant, resulting in cellular exposure to this stimulant over the entire cell surface in the medium. Therefore, it is likely that the more activated caspases diffuse in parallel, leading to the appearance of the more homogeneous signal. To settle this issue and spatiotemporally understand the real status of the apoptotic signal transmission, it will require the analysis of a solid directional apoptotic signal arising from a well-defined start point.

In summary, we developed the dual-FRET system with a triple fusion fluorescent protein to temporally monitor the activation of plural caspases. With this system, we were able to visualize the progress of caspase activation was determined at the single-cell level. Also, for the first time, functional in vitro reconstitution of the caspase cascade using only recombinant proteins was able to exactly reproduce the signaling cascade in the cells. Finally, we verified the biological significance of the positive feedback loop and the diffusion process leading to full CASP8 activation.

## Materials and Methods

### Generation of Plasmid Constructs

To generate a FRET-based biosensor molecule, CYR83, consisting of three fluorescent proteins seCFP, Venus [10], and mRFP1 [11] and carrying two different caspase recognition sequences IETD and DEVD in the linker portions, we fused seCFP, Venus, and mRFP1 cDNAs with the liker DNA fragments. Two linkers carry the sequences encoding SSSELSGIETDGTSGSEF and SSSELSGDEVDGTSGSEF, respectively. To generate two CYR83 variants, CYR83(IETA) and CYR83(DEVA), the aspartic acid residues in the IETD or DEVD sequences were replaced with alanine, by insertion of a PCR-amplified DNA fragment containing the variant sequence. To express in mammalian cells, DNA fragments encoding CYR83 and its variants were cloned into an expression vector, pCAGGS [41].

To prepare recombinant proteins, plasmid constructs were generated to express in *E. coli*. For preparation of recombinant CYR83, seCFP, Venus and mRFP1 proteins, DNA fragments encoding these molecules were fused with a His-tag at N-terminus and cloned into pRSET (Invitrogen, Carlsbad, CA). To prepare a fusion protein, SCAT8.1, a DNA fragment carrying seCFP, IETD sequence and Venus was cloned into pcDNA4/HisMAx (Invitrogen). SCAT3.1 was generated as described previously [19]. To produce procaspases and FKBP-CASP8 proteins with the cell-free system, human CASP3 and CASP6 cDNAs and an FKBP-CASP8 fusion gene [18] were cloned into the pEU vector, which is customized for protein synthesis [17]. To produce pro-CASP3 mutant, a protease-deficient CASP3 mutant cDNA was generated by replacing the cysteine residue in the QACRG active site of the protease region with serine by exchange of the PCR-amplified DNA fragment containing the mutant sequence and cloned into pEU.

To generate a membrane-bound SCAT8, GsSCAT8, a DNA fragment corresponding to N-terminal 20 amino acids (MLCCMRRTKQVEKNDEDQKI) of growth associated protein 43 (Gap43) was inserted at the 5′ end of SCAT8.2. To establish the red fluorescent labeled-cells, the plasmid pCAGGS-mCherry was generated by inserting a PCR-amplified DNA fragment encoding mCherry [42] into pCAGGS. To establish the stable neomycin-resistant cell lines, we constructed the expression plasmid pME18S-Neo by inserting the neomycin resistance gene into pME18S [43].

To downregulate CASP8 proteins in cells, the plasmid pCSIIU6Tet-shCASP8-Neo inducing the expression of short hairpin RNA (shRNA) against *CASP8* mRNA was generated. A DNA fragment carrying target sequences, shCASP8∶5′-TCTTCCGAATTAATAGACT-3′ was synthesized and cloned into pCSII-U6Tet-Neo [44]. To express a fusion protein, CASP8CS/mKikGR, in cells, the plasmid pCMV-hCASP8CS/mKikGR was generated. Briefly, human *CASP8* cDNA encoding a protease-deficient CS mutant was generated by replacing the cysteine residue in the active site of the protease domain with serine, by exchange of a PCR-amplified DNA fragment containing the mutant sequence. Then this mutant cDNA was joined with both a DNA fragment encoding triple GGGS repeat sequence [45] and a cDNA encoding a photoconvertible protein, mKikGR [22], and cloned into pIREShyg2 (Clontech, Mountain View, CA). To overexpress wild-type CASP6 or its mutant CASP6CS, the plasmid constructs, which have previously been generated [20], were used.

### Cell Culture and Transfection

Human cervical carcinoma HeLa cells and embryonic kidney 293 (HEK293) cells derived from ATCC were cultured in Dulbecco’s Modified Eagle’s medium with 10% fetal calf serum (FCS). The CASP8-knockdown HeLa cells were established by transfection with the plasmid pCSIIU6Tet-shCASP8-Neo and selection in the presence of 1.2 mg/ml of G418 (Nacalai Tesque, Kyoto, Japan). The stable transfectant of mouse WR19L lymphoma, WD4, which expresses human FasL [21], was provided by S. Nagata (Kyoto University) and cultured in RPMI 1640 medium supplemented with 10% FCS. Transfection of the plasmid DNAs into HeLa cells was carried out using Lipofect2000 (Invitrogen) according to manufacturers’ instructions. To label WD4 cells with mCherry, they were transfected with 15 µg of pCAGGS-mCherry and pME18S-Neo by electroporation using a Gene Pulser (Bio-Rad Laboratories, Hercules, CA) and selected in the presence of 2 mg/ml of G418.

### Bioimaging with a Fluorescent Microscopy

Cells transfected with plasmid constructs were plated on a 35-mm glass-bottomed dish (Asahi Technoglass Co., Tokyo, Japan) and maintained in growth medium for 1 day. Prior to induction of apoptosis, media was replaced with PBS containing 1 mM CaCl_2_, 0.5 mM MgCl_2_ and 10% FCS and then either treated with 200 ng/ml of agonistic anti-Fas antibody (CH-11, MBL, Nagoya, Japan) in the presence of 5 µg/ml cycloheximide (CHX) or UV-irradiated at 200 J/m^2^ using a UV linker (FS800, Funakoshi, Tokyo, Japan). During monitoring, the cells were plated in a heated chamber (MI-IBC, Olympus Co., Tokyo, Japan). Fluorescence images were acquired with an interlined charge-coupled device (CCD) camera (CoolSNAP HQ, Roper Scientific, Tucson, AZ) under an inverted microscope (DMIRE2, Leica Microsystems, Wetzlar, Germany) controlled by MetaFluor software (Universal Imaging, Media, PA). The D425/40x, HQ500/20x and HQ545/30x excitation filters and D480/40 m, HQ535/30 m and HQ620/60 m emission filters (Chroma Technology Corp. Rockingham, VT) and a glass plate with a refractive index of 0.04 instead of a dichroic mirror were used for the detection of the fluorescence under the control of a filter changer (MAC5000, Ludl Electronic Products Ltd., Hawthorne, NY). That is, based on the five multi-channel ways by combinations with these excitation and emission filters, we took images of each of the three fluorophores and two “raw” FRET images: (1) seCFP using D425/40x & D480/40 m filters, (2) Venus using HQ500/20x & HQ535/30 m filters, (3) mRFP1 using HQ545/30x & HQ620/60 m filter, (4) seCFP**→**Venus using D425/40x & HQ535/30 m filters, and (5) Venus**→**mRFP1 using HQ500/20x & HQ620/60 m filters. To monitor the FRET ratio of GsSCAT8, the fluorescence of both seCFP and seCFP**→**Venus were acquired in single cells as well.

To monitor the diffusion of CASP8CS/mKikGR, HeLa cells transiently expressing CASP8CS/mKikGR were plated on a 35-mm glass-bottomed dish and cultured for 1 day. Images were acquired on a confocal microscope, FV1000-D (Olympus) equipped with a PlanApo N 60x 1.42 numerical aperture oil objective (Olympus), multi-Argon ion laser and laser diodes. To photoswitch the fluorescence of mKikGR from the green to red form, a 405 nm laser was irradiated for 100 msec. The green fluorescence signals were excited 488 nm laser line and detected at the 500–545 nm wavelength range. The red fluorescence signals were excited 559 nm laser line and detected at the 570–670 nm wavelength range. The fluorescence ratio and intensity of a region of interest were calculated by using the FV10-ASW software (Olympus).

### Preparation of Recombinant Proteins

For preparation of recombinant proteins, the plasmid carrying CYR83 was transfected into *E. coli* JM109 (DE3) (Promega, Madison, WI); after 3 days culture at 20°C, transfectants were lysed in B-PER II Bacterial Protein Extraction Reagent (Pierce, Rockford, IL). Recombinant CYR83 proteins were then purified using nickel-NTA column chromatography (Qiagen, Valencia, CA). Similarly, His-tagged fluorescent proteins, seCFP, Venus and mRFP1 and FRET-based biosensor proteins, SCAT3.1 and SCAT8.1 were also purified from *E. coli* cell lysates after transfection of plasmid constructs.

### Preparation of Pro-caspases, and FKBP-CASP8 with a Cell-free System

Recombinant procaspases, fusion protein and caspase mutant were produced using a cell-free system as previously described [17]. Briefly, pro-CASP3, pro-CASP6, FKBP-CASP8 and pro-CASP3 mutant were transcribed from the SP6 promoter of the pEU expression vector; the resulting transcripts were translated with wheat embryo extracts (WEPRO-1240, CellFree Sciences, Matsuyama, Japan). Recovery of reactive products was confirmed by SDS-PAGE and following by immunoblot analysis with anti-caspase antibodies.

### Spectral Analysis

Five hundred nanogram of recombinant CYR83 proteins was incubated with either active CASP8 (0.5 unit) or CASP3 (0.5 unit) in the protease assay solution [10% sucrose, 50 mM PIPES (pH7.2), 100 mM NaCl, 10 mM DTT, 0.1% CHAPS, 1 mM EDTA] at 37°C for 2 h. Prior to and after incubation, the emission spectra of the mixture were measured at an excitation wavelength of either 440 nm or 500 nm using a fluorescence spectrophotometer (SPEX Fluorolog-3, HORIBA JOBIN YVON SAS, Longjumeau, France).

### Fluorescent Image Analyses

For the *in vitro* cleavage assay, recombinant active caspases, human CASP3 (code no. E001), human CASP6 (code no. BV-1086-2), human CASP8 (code no. BV-10888-2) and human CASP9 (code no. BV-1089-2) were purchased from MBL. Processing activities of active caspases (ea. 1 unit) to CYR83 (1 µg) were examined in protease assay solution at 37°C for 2 h. The reaction was stopped by adding sample buffer, and samples were resolved by SDS-PAGE without heat denaturation together with FITC-labeled molecular weight markers (SP-0130, APRO Life Science Institute, Tokushima, Japan) and 300 ng of recombinant seCFP, Venus and mRFP1. The fluorescence in the gel was directly analyzed using an imaging analyzer (Typhoon 9410, GE Healthcare, Buckinghamshire, United Kingdom) with the ImageQuant software. For the detection of the fluorescence intensity of the cleaved peptide fragments, we used lasers at 457 nm, 532 nm and 633 nm for excitation and filters of 526SP, 555BP20 and 610BP30 specific for emission spectrum. To analyze the proteolytic processing profiles of the SCAT3.1 and SCAT8.1 probes, various reaction mixtures containing either SCAT3.1 or SCAT8.1 combined with several pro-caspases were incubated at 37°C for 2 h, and the cleaved products were detected by gel electrophoresis and scanning using the imaging analyzer. The proteolytic processing patterns of fluorescent indicator proteins expressed in HeLa cells undergoing apoptosis were also examined in the same procedure.

### Immunoblot Analyses

For the detection of the full-length and processed forms of caspases in the *in vitro* assays, mixtures were resolved by SDS-PAGE and followed by immunoblotting with anti-CASP3, anti-CASP6 and anti-CASP8 antibodies (Cell Signaling Technology, Danvers, MA). After incubation with HRP-conjugated anti-mouse or anti-rabbit IgG antibodies (Cell Signaling Technology), proteins were visualized with Immobilon^TM^ Western (Millipore Corporation, Billerica, MA) using an image analyzer (LAS-3000, FUJIFILM, Tokyo, Japan).

To confirm the knockdown efficiency of endogenous CASP8 in HeLa cells by RNA interference, cell lysates were resolved by SDS-PAGE and followed by immunoblotting with anti-CASP8 and anti-actin (MAB1501R, Chemicon International Inc., Temecula, CA) antibodies, respectively. The fusion protein, CASP8CS/mKikGR in transfected cells was examined by the same procedure. In addition, the processing profiles of endogenous CASP3 and PARP and exogenous SCAT8.1 in cells undergoing apoptosis were examined by immunoblotting with anti-CASP3, anti-PARP (#9542, Cell Signaling Technology) and anti-GFP (#598, MBL) antibodies. Quantification of the immunoblot bands was performed with the MultiGauge software (FUJIFILM).

### Construction of a Mathematical Model of Activated CASP8 Diffusion

The diffusion of activated CASP8 is analyzed by using the one-dimensional diffusion model. We assumed that the activated CASP8 is released from the origin and diffuse in one dimension. The reaction term such as the activation of CASP8 is neglected (i.e. the density of CASP8 is determined by the diffusion from the stimulus point only). We also assumed that the stimulus point is at the origin with the cell size *L*. Then by denoting the density of activated CASP8 as *u*(*x*,*t*), where *x* (0 ≤ *x* ≤ *L*) and *t* (0 ≤ *t*) denote the coordinate and time, respectively. The diffusion model is formulated by the following one-dimensional diffusion equation.
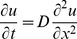
Where *D* is the diffusion coefficient of activated CASP8. For the boundary conditions, it is assumed that, depending on the stimulus we have a specific value of activated CASP8 at *x* = 0 and a reflection boundary at *x* = *L*.



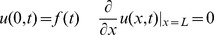



Temporal stimulus at the origin (*x = *0) is denoted as *f*(*t*) which is given as follows.
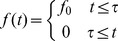



Here, *f*_0_ is the temporal value of activated CASP8 at the origin and *τ* is the duration time of this temporal stimulus. Given the above boundary conditions and the initial condition *u*(*x*,0) = 0, one-dimensional diffusion equation is simulated using a finite difference method.

### Statistical Analysis

The statistical difference between the two groups was examined by Student’s *t*-test.

## Supporting Information

Figure S1 **Imaging of a FRET-based biosensor, CYR83 incubated with active caspases in the gel.** (A-C) Products generated from CYR83 by processing with active CASP8 and CASP9. Recombinant CYR83 (1 µg) was incubated with active CASP8 (1 unit) or CASP9 (1 unit) for 0–120 min and resolved by SDS-PAGE. For the detection of the fluorescence of seCFP (A), Venus (B) and mRFP1 (C), the gel was repeatedly scanned with three types of laser (457 nm, 532 nm and 633 nm) and emission filters (526SP, 555BP20 and 610BP30) using an imaging analyzer. (D-F) Products generated from CYR83 by processing with active CASP3. Recombinant CYR83 (1 µg) was incubated with active CASP3 (1 unit) at indicated times and resolved by SDS-PAGE. For the detection of the fluorescence of seCFP (D), Venus (E) and mRFP1 (F), the gel was repeatedly scanned as described for (A-C). Lower-case characters indicate full length CYR83 (a) and the increased peptide fragments (b-f) during incubation with active caspases. Abbreviations; x, unidentifiable peptide fragment; M, FITC-conjugated molecular weight markers; C, seCFP; V, Venus; R, mRFP1.
(TIF)

Figure S2 **Biochemical analyses of active CASP8 against for CYR83.** (A) Comparison of the catalytic activities of CASP8 and CASP9. By counting the fluorescent intensities of seCFP products processed from CYR83 (1 µg) as shown in Figure 1E, the catalytic activities of both active CASP8 (open circle) and CASP9 (closed circle) (ea 1 unit) were determined. The recombinant seCFP protein was used as a standard control. (B) Active CASP8 was incubated without and with 0.25, 0.5, 1, 2 or 4 µg of CYR83 for 30 min. After resolution of the reaction mixture by electrophoresis, the fluorescent intensity of seCFP products processed from CYR83 was measured and plotted on a diagram. (C) Estimation of the Vmax and Km of CASP8 by Lineweaver-Burk plot. Here, data from B are plotted as reciprocal values. According to the Lineweaver-Burk formula, Vmax = 1/1.1063 = 0.90 (ng/U/min) and Km = −1/(−1.1063/2.2891) = 2.06 (µg) were estimated.
(TIF)

Figure S3 **Adjusting of time course in caspase activation.** (A) The imaging pattern of SCAT3-expressing HeLa cells undergoing apoptosis. On two cells numbered as #1 and #2, large and small circles (Spot A and B) were set up for monitoring of FRET and the detection of shrinkage, respectively. Pseudo colors indicate the emission ratio of calculated fluorescent intensity passing through 535 nm and 480 nm filters, and were varied from 2.2 to 1.0 during monitoring. An arrow indicates the moment that each cell withdrew from a Spot B on the way to scanning. (B) A time course of the emission ratio with SCAT3 in the single cells. The fluorescence in a ‘Spot A’ shown in (A) was acquired through filters, calculated the emission ratio and converted into a diagram. Red and blue arrows indicate the time point that cells withdrew from ‘Spot B’ due to shrinkage. (C) The adjusted graphic pattern. Graphic data shown in (B) were adjusted by converting the moment when cells went away from a scanning spot to time zero.
(TIF)

Figure S4 **Imaging profiles of CASP8 and CASP3 activation associated with apoptosis.** (A, B) Serial fluorescence ratio images of dying HeLa cells using a FRET-based CYR83. For the detection of CASP8 (A) and CASP3 (B) activation, three cells in the same field were monitored through filters as described in the Materials and Methods. Numbers indicate time after taking the first image.
(TIF)

Figure S5 **Analyses of the proteolytic processing profiles of CYR83 and its variants in cells undergoing apoptosis.** (A-C) Detection of the cleaved peptide fragments from CYR83 and its variants. Cell extracts of HeLa cells expressing either CYR83, CYR83(IETA) or CYR83(DEVA) were prepared at indicated times after Fas ligation and resolved by SDS-PAGE. For the detection of the fluorescence of seCFP (A), Venus (B) and mRFP1 (C), the gel was repeatedly scanned with three types of laser (457 nm, 532 nm and 633 nm) and emission filters (526SP, 555BP20 and 610BP30) using an imaging analyzer. (D) Immunoblot analyses of cell extracts prepared from HeLa cells expressing either CYR83, CYR83(IETA) or CYR83(DEVA). Endogenous PARP and actin were examined with indicated specific antibodies. (E-G) Detection of the cleaved peptide fragments from CYR83 and CYR83(IETA). Cell extracts of HeLa cells expressing either CYR83 or CYR83(IETA) were prepared at indicated times after UV-irradiation and resolved by SDS-PAGE. The fluorescence of seCFP (E), Venus (F) and mRFP1 (G) in the gel was repeatedly scanned. (H) Immunoblot analyses of cell extracts prepared from CYR83- or CYR83(IETA)-expressing HeLa cells after UV-irradiation. The proteolytic processing pattern of endogenous PARP was examined by immunoblotting. Lower-case characters shown in (A-C, E-G) indicate a full-length (a) and the cleaved peptide fragments (b-f), corresponding to those shown in Figure 1E. An arrow indicates intact PARP while a white arrowhead identifies the cleaved fragments (D, H).
(TIF)

Figure S6 **In vitro cleavage assays of SCAT8.1 with recombinant caspases.** (A) Fluorescence image analysis on the proteolytic processing profile of the SCAT8.1 probe. Various reaction mixtures containing SCAT8.1 combined with several caspases were incubated at 37°C for 6 h, and the cleaved products were resolved by SDS-PAGE and visualized by scanning of the fluorescence in the gel. The arrow and arrowheads indicate the full-length and cleaved form of the probe, respectively. (B) Immunoblot analyses on the cleavage of SCAT8.1 and procaspases (pro-CASP3, pro-CASP6 and FKBP-CASP8) in the reaction mixtures. Samples were analyzed by SDS-PAGE following immunoblotting with indicated specific antibodies. The arrow and arrowheads indicate the full-length and the cleaved form of proteins examined, respectively. Abbreviations: C, seCFP; W, wild-type pro-CASP3; M, the protease-defect pro-CASP3 mutant.
(TIF)

Movie S1 **Dual real-time images displaying caspase activation.** IETDase (left half) and DEVDase (right half) activities specific for a FRET-based CYR83 biosensor in transfected cells, analyzed by microscopy. Transfectants expressing CYR83 were treated with anti-Fas antibody and CHX and their fluorescence images were captured every 15 sec for 2 h under a fluorescent microscope (DMIRE2, Leica Microsystems) equipped with a HCX PL Apo 64×, 1.4 NA oil-immersion objective (Leica Microsystems), using a CCD camera through the specified filters [Excitation (Ex) 425 nm/Emission (Em) 480 nm for seCFP, Ex 500 nm/Em 535 nm for Venus, Ex 545 nm/Em 620 nm for mRFP1, Ex 425 nm/Em 535 nm for seCFP→Venus, Ex 500 nm/Em 620 nm for Venus→mRFP1]. Finally, the serial fluorescence images were displayed by pseudo color based on calculated fluorescent intensity.
(MPG)

Movie S2 **Transmission of CASP8 activation associated with apoptosis.** The fluorescence intensity of HeLa cells expressing a membrane-bound FRET-based biosensor, GsSCAT8, was captured every 30 sec for 100 min under a fluorescent microscope. The serial ratio images were displayed by pseudo color based on calculated the fluorescence intensity.
(MPG)
