# Supplementary figures and images for: In Vivo Imaging of Hierarchical Spatiotemporal Activation of Caspase-8 during Apoptosis
Source: PLoS One. 2012 Nov 21;7(11):e50218. doi: 10.1371/journal.pone.0050218 (PMC3503975; doi:10.1371/journal.pone.0050218)

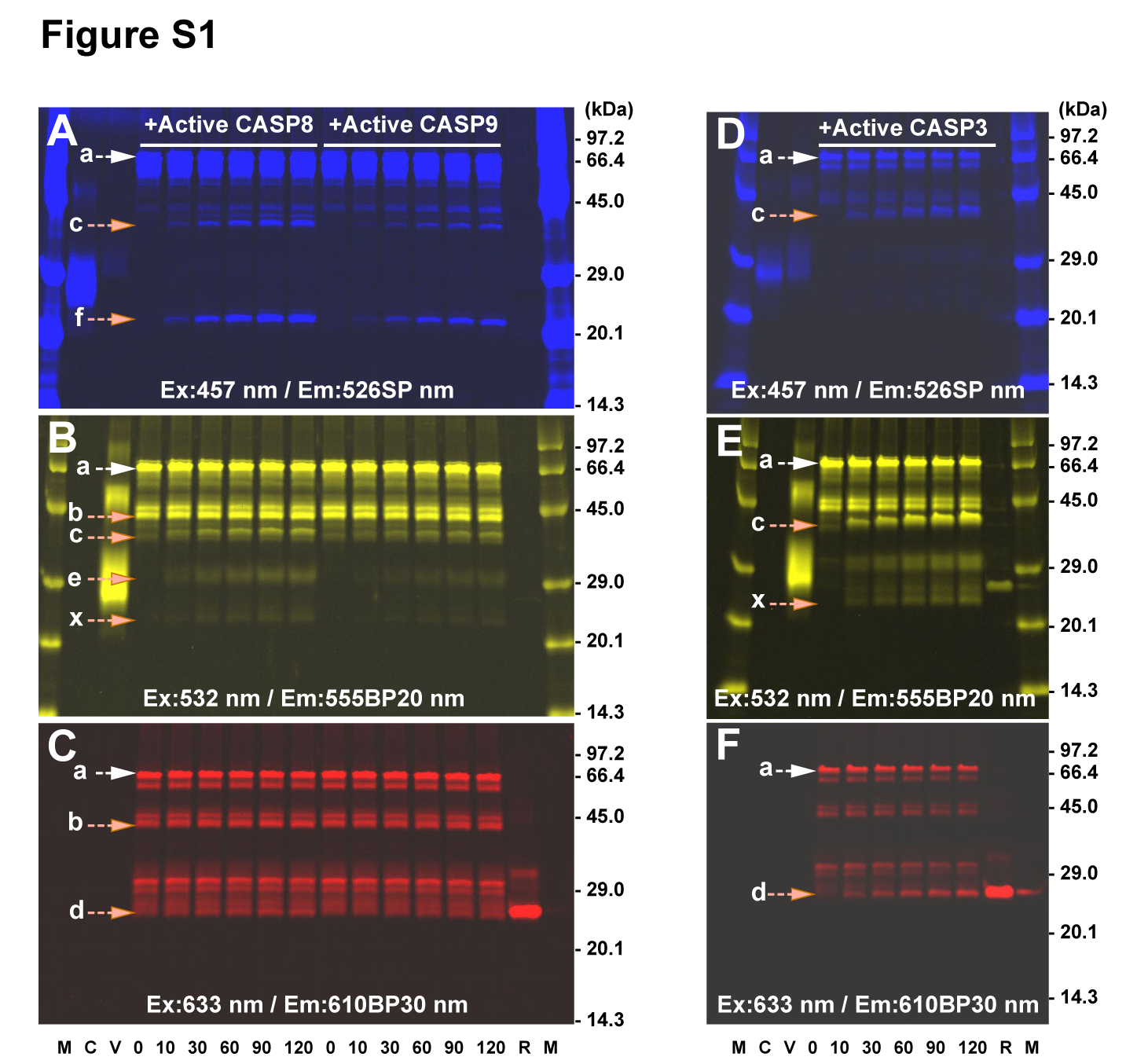

Supplement: Figure S1 — Imaging of a FRET-based biosensor, CYR83 incubated with active caspases in the gel. (A-C) Products generated from CYR83 by processing with active CASP8 and CASP9. Recombinant CYR83 (1 µg) was incubated with active CASP8 (1 unit) or CASP9 (1 unit) for 0–120 min and resolved by SDS-PAGE. For the detection of the fluorescence of seCFP (A), Venus (B) and mRFP1 (C), the gel was repeatedly scanned with three types of laser (457 nm, 532 nm and 633 nm) and emission filters (526SP, 555BP20 and 610BP30) using an imaging analyzer. (D-F) Products generated from CYR83 by processing with active CASP3. Recombinant CYR83 (1 µg) was incubated with active CASP3 (1 unit) at indicated times and resolved by SDS-PAGE. For the detection of the fluorescence of seCFP (D), Venus (E) and mRFP1 (F), the gel was repeatedly scanned as described for (A-C). Lower-case characters indicate full length CYR83 (a) and the increased peptide fragments (b-f) during incubation with active caspases. Abbreviations; x, unidentifiable peptide fragment; M, FITC-conjugated molecular weight markers; C, seCFP; V, Venus; R, mRFP1. (TIF) [file pone.0050218.s001.tif]

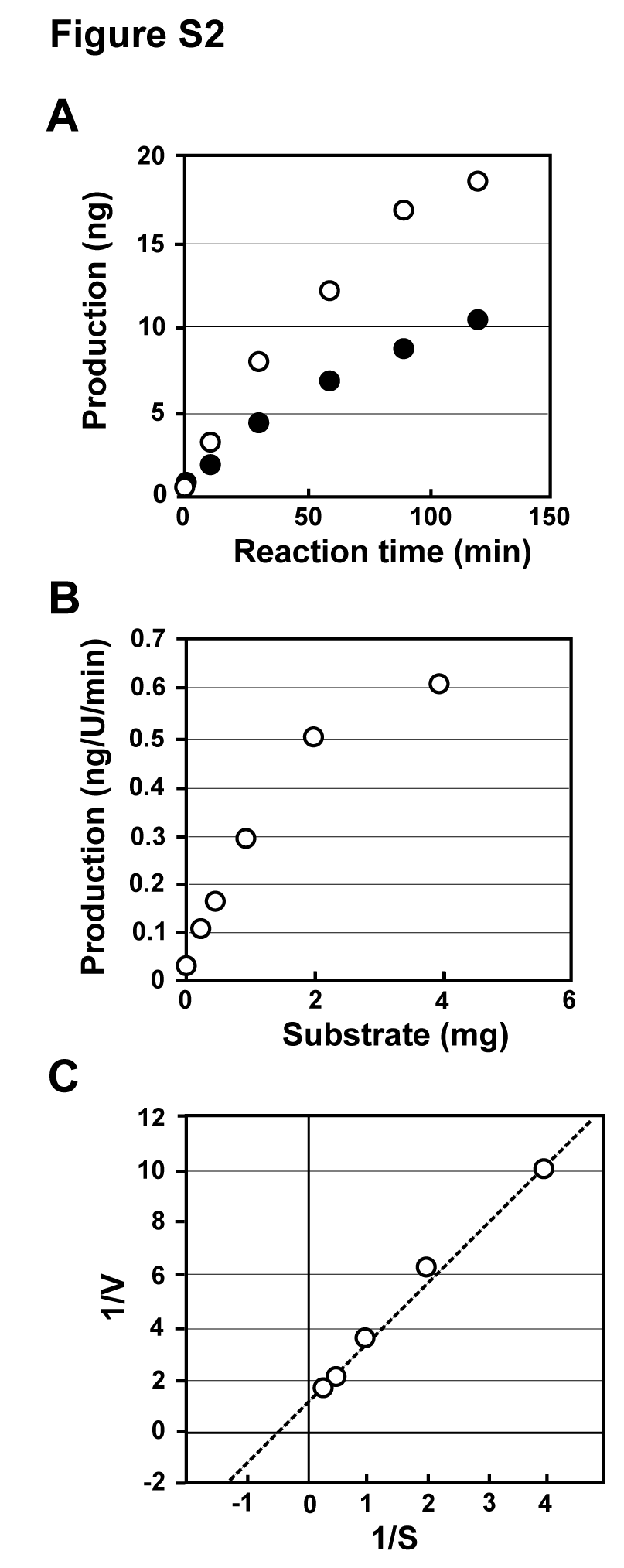

Supplement: Figure S2 — Biochemical analyses of active CASP8 against for CYR83. (A) Comparison of the catalytic activities of CASP8 and CASP9. By counting the fluorescent intensities of seCFP products processed from CYR83 (1 µg) as shown in Figure 1E, the catalytic activities of both active CASP8 (open circle) and CASP9 (closed circle) (ea 1 unit) were determined. The recombinant seCFP protein was used as a standard control. (B) Active CASP8 was incubated without and with 0.25, 0.5, 1, 2 or 4 µg of CYR83 for 30 min. After resolution of the reaction mixture by electrophoresis, the fluorescent intensity of seCFP products processed from CYR83 was measured and plotted on a diagram. (C) Estimation of the Vmax and Km of CASP8 by Lineweaver-Burk plot. Here, data from B are plotted as reciprocal values. According to the Lineweaver-Burk formula, Vmax = 1/1.1063 = 0.90 (ng/U/min) and Km = −1/(−1.1063/2.2891) = 2.06 (µg) were estimated. (TIF) [file pone.0050218.s002.tif]

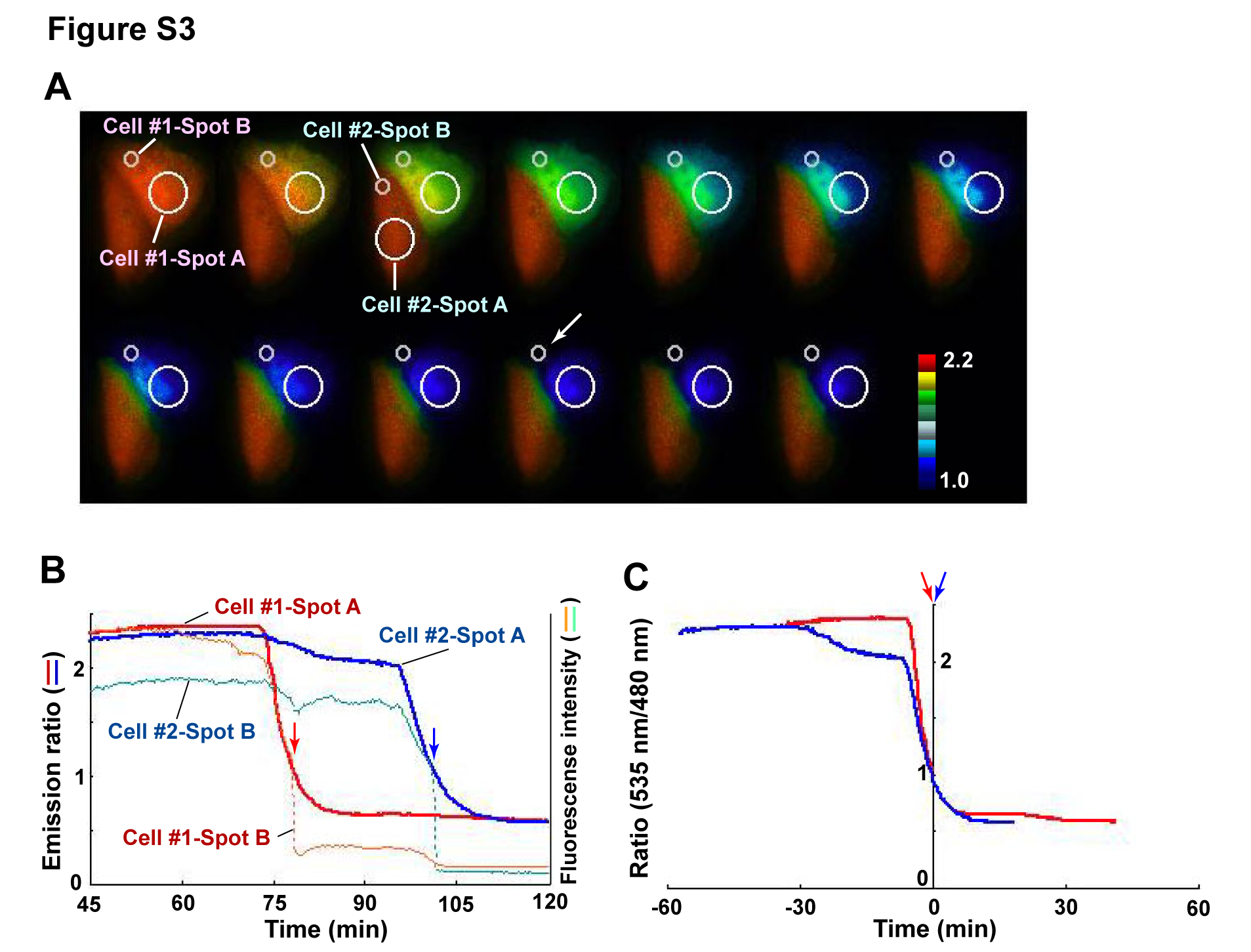

Supplement: Figure S3 — Adjusting of time course in caspase activation. (A) The imaging pattern of SCAT3-expressing HeLa cells undergoing apoptosis. On two cells numbered as #1 and #2, large and small circles (Spot A and B) were set up for monitoring of FRET and the detection of shrinkage, respectively. Pseudo colors indicate the emission ratio of calculated fluorescent intensity passing through 535 nm and 480 nm filters, and were varied from 2.2 to 1.0 during monitoring. An arrow indicates the moment that each cell withdrew from a Spot B on the way to scanning. (B) A time course of the emission ratio with SCAT3 in the single cells. The fluorescence in a ‘Spot A’ shown in (A) was acquired through filters, calculated the emission ratio and converted into a diagram. Red and blue arrows indicate the time point that cells withdrew from ‘Spot B’ due to shrinkage. (C) The adjusted graphic pattern. Graphic data shown in (B) were adjusted by converting the moment when cells went away from a scanning spot to time zero. (TIF) [file pone.0050218.s003.tif]

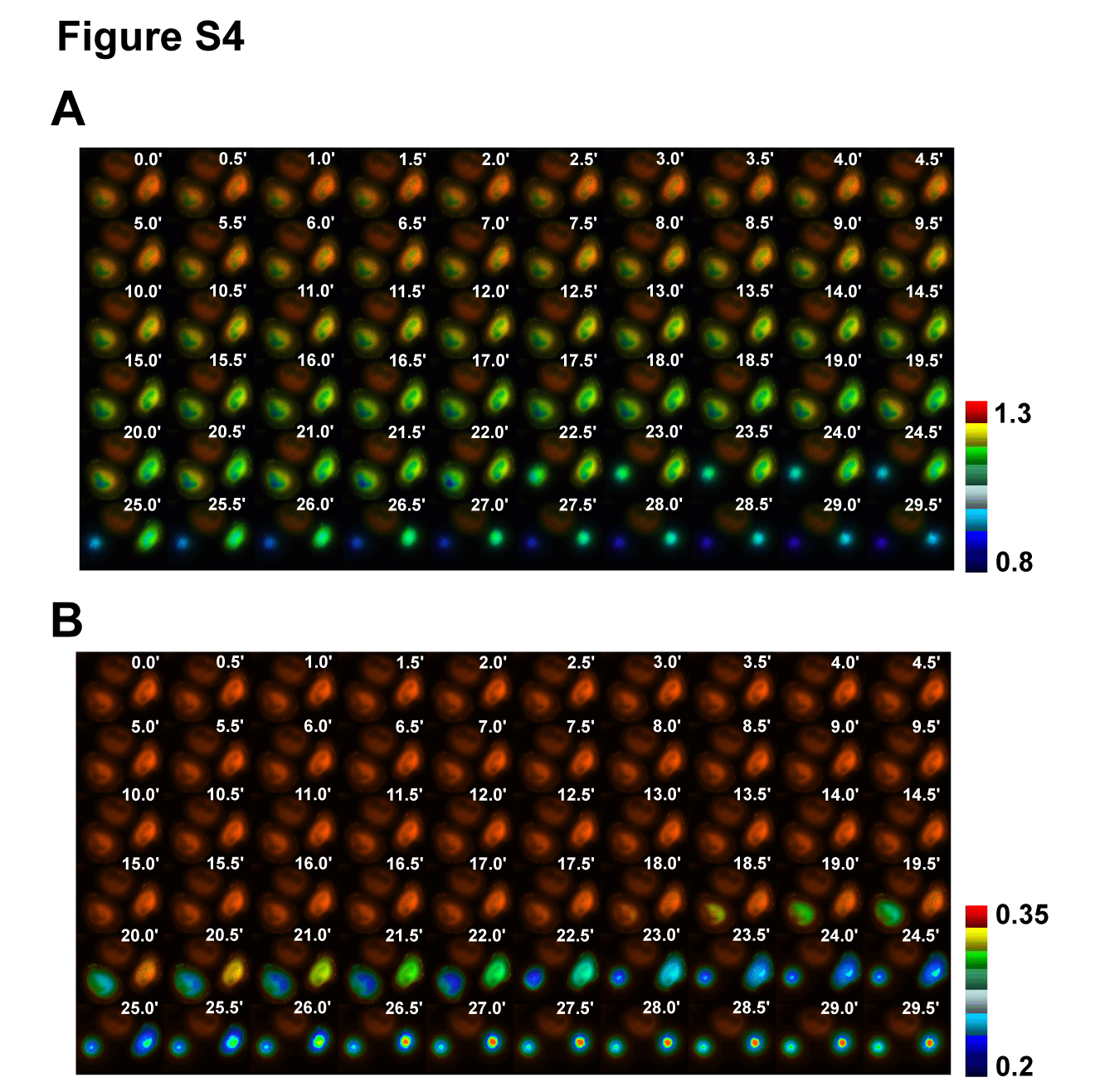

Supplement: Figure S4 — Imaging profiles of CASP8 and CASP3 activation associated with apoptosis. (A, B) Serial fluorescence ratio images of dying HeLa cells using a FRET-based CYR83. For the detection of CASP8 (A) and CASP3 (B) activation, three cells in the same field were monitored through filters as described in the Materials and Methods. Numbers indicate time after taking the first image. (TIF) [file pone.0050218.s004.tif]

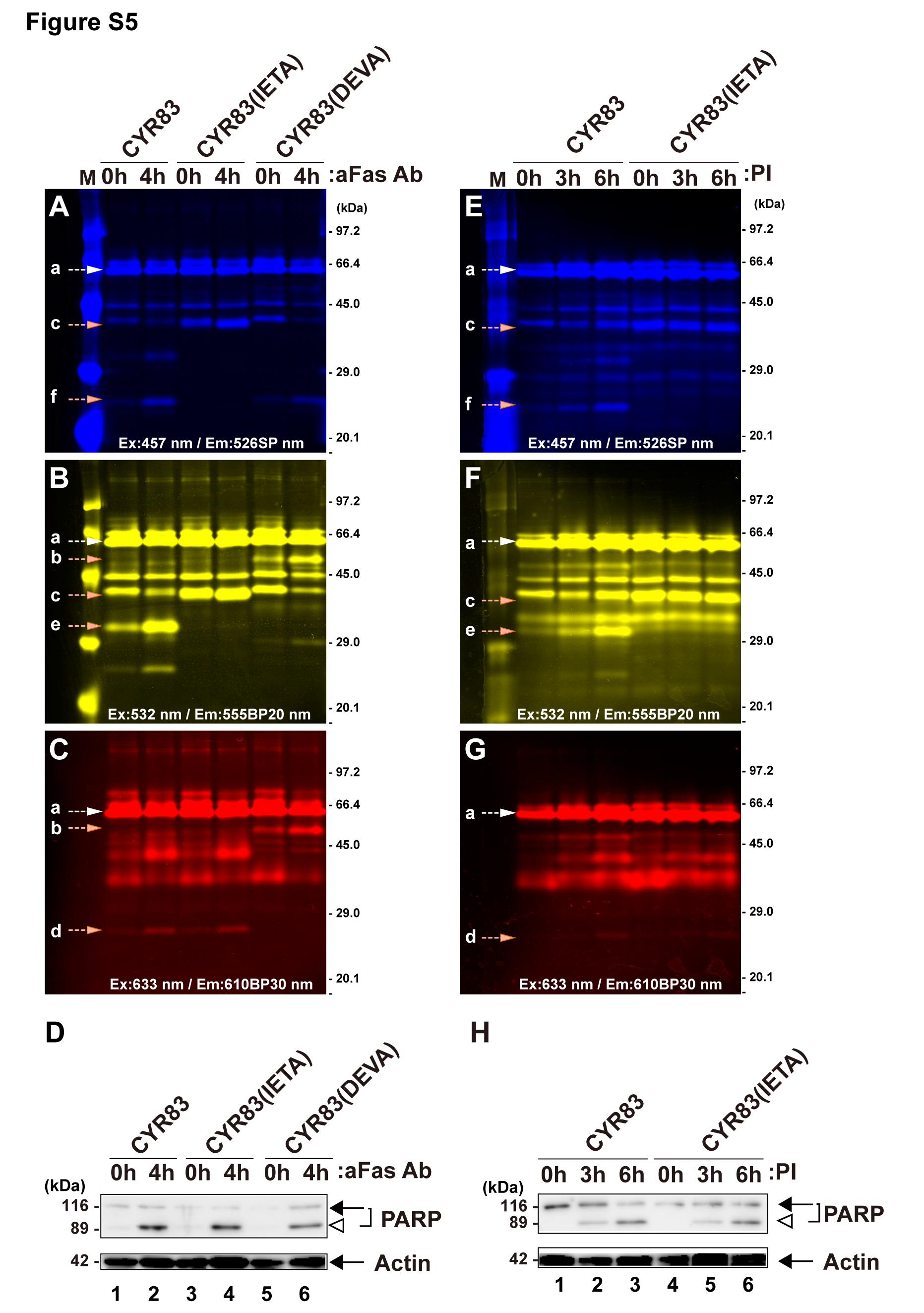

Supplement: Figure S5 — Analyses of the proteolytic processing profiles of CYR83 and its variants in cells undergoing apoptosis. (A-C) Detection of the cleaved peptide fragments from CYR83 and its variants. Cell extracts of HeLa cells expressing either CYR83, CYR83(IETA) or CYR83(DEVA) were prepared at indicated times after Fas ligation and resolved by SDS-PAGE. For the detection of the fluorescence of seCFP (A), Venus (B) and mRFP1 (C), the gel was repeatedly scanned with three types of laser (457 nm, 532 nm and 633 nm) and emission filters (526SP, 555BP20 and 610BP30) using an imaging analyzer. (D) Immunoblot analyses of cell extracts prepared from HeLa cells expressing either CYR83, CYR83(IETA) or CYR83(DEVA). Endogenous PARP and actin were examined with indicated specific antibodies. (E-G) Detection of the cleaved peptide fragments from CYR83 and CYR83(IETA). Cell extracts of HeLa cells expressing either CYR83 or CYR83(IETA) were prepared at indicated times after UV-irradiation and resolved by SDS-PAGE. The fluorescence of seCFP (E), Venus (F) and mRFP1 (G) in the gel was repeatedly scanned. (H) Immunoblot analyses of cell extracts prepared from CYR83- or CYR83(IETA)-expressing HeLa cells after UV-irradiation. The proteolytic processing pattern of endogenous PARP was examined by immunoblotting. Lower-case characters shown in (A-C, E-G) indicate a full-length (a) and the cleaved peptide fragments (b-f), corresponding to those shown in Figure 1E. An arrow indicates intact PARP while a white arrowhead identifies the cleaved fragments (D, H). (TIF) [file pone.0050218.s005.tif]

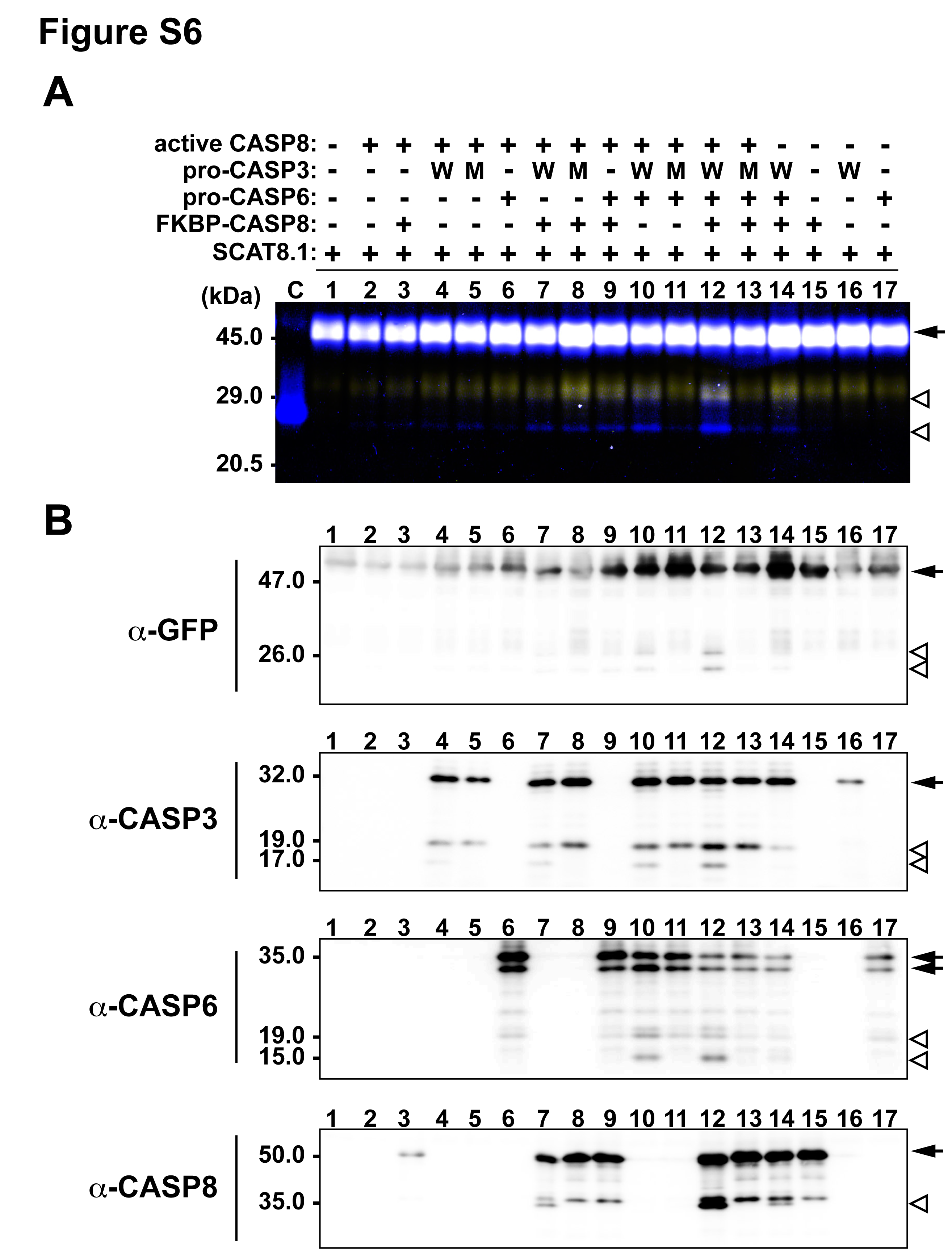

Supplement: Figure S6 — In vitro cleavage assays of SCAT8.1 with recombinant caspases. (A) Fluorescence image analysis on the proteolytic processing profile of the SCAT8.1 probe. Various reaction mixtures containing SCAT8.1 combined with several caspases were incubated at 37°C for 6 h, and the cleaved products were resolved by SDS-PAGE and visualized by scanning of the fluorescence in the gel. The arrow and arrowheads indicate the full-length and cleaved form of the probe, respectively. (B) Immunoblot analyses on the cleavage of SCAT8.1 and procaspases (pro-CASP3, pro-CASP6 and FKBP-CASP8) in the reaction mixtures. Samples were analyzed by SDS-PAGE following immunoblotting with indicated specific antibodies. The arrow and arrowheads indicate the full-length and the cleaved form of proteins examined, respectively. Abbreviations: C, seCFP; W, wild-type pro-CASP3; M, the protease-defect pro-CASP3 mutant. (TIF) [file pone.0050218.s006.tif]
